# Current Drug Repurposing Strategies for Rare Neurodegenerative Disorders

**DOI:** 10.3389/fphar.2021.768023

**Published:** 2021-12-21

**Authors:** Sweta Shah, Marc Marie Dooms, Sofia Amaral-Garcia, Mariana Igoillo-Esteve

**Affiliations:** ^1^ Faculty of Medicine, Université Libre de Bruxelles, Brussels, Belgium; ^2^ University Hospitals Leuven, Leuven, Belgium; ^3^ Joint Research Center of the European Commission, Brussels, Belgium; ^4^ ULB Center for Diabetes Research, Université Libre de Bruxelles, Brussels, Belgium

**Keywords:** drug repurposing, wolfram syndrome, amyotrophic lateral sclerosis, Huntington’s disease, Friedreich’s ataxia, European Medicines Agency (EMA), Food and Drug Administration (FDA, US), orphanet

## Abstract

Rare diseases are life-threatening or chronically debilitating low-prevalent disorders caused by pathogenic mutations or particular environmental insults. Due to their high complexity and low frequency, important gaps still exist in their prevention, diagnosis, and treatment. Since new drug discovery is a very costly and time-consuming process, leading pharmaceutical companies show relatively low interest in orphan drug research and development due to the high cost of investments compared to the low market return of the product. Drug repurposing–based approaches appear then as cost- and time-saving strategies for the development of therapeutic opportunities for rare diseases. In this article, we discuss the scientific, regulatory, and economic aspects of the development of repurposed drugs for the treatment of rare neurodegenerative disorders with a particular focus on Huntington’s disease, Friedreich’s ataxia, Wolfram syndrome, and amyotrophic lateral sclerosis. The role of academia, pharmaceutical companies, patient associations, and foundations in the identification of candidate compounds and their preclinical and clinical evaluation will also be discussed.

## Introduction

### Rare Neurodegenerative Diseases

Rare neurodegenerative diseases are low-prevalent, life-threatening, or chronically debilitating disorders, caused by pathogenic mutations in a single gene or by particular environmental insults (e.g., pesticides, metals, air pollution, endotoxins, and prions, among others), triggering progressive neuronal dysfunction and loss of specific groups of neurons (Matilla-Duenas et al., 2017). Depending on the disease etiology, distinct parts of the central nervous system may be affected, resulting in impaired motor and cognitive function with a significant impact on the quality of life of the affected individuals (Matilla-Duenas et al., 2017). The prevalence threshold defining a disease as rare largely varies between countries, but disorders with a prevalence of five cases or less per 10,000 individuals according to the European Union (EU) are designated as such (Nguengang Wakap et al., 2020). In the last few years, the advances in next-generation sequencing (NGS) have importantly accelerated the identification of disease-causing genes. Indeed, the Online Mendelian Inheritance in Man database (https://omim.org/statistics/geneMap) of July 2021 reports 4473 genes with phenotype-causing mutations resulting in neurological and non-neurological disorders. Despite the advances in next generation sequencing (NGS), data analysis, and other technologies that importantly contribute to identify the mutated genes and understand the biology of rare diseases and their underlying pathogenic mechanisms, the diagnosis, treatment, and availability of therapeutics for these pathologies are still very limited (Fernandez-Marmiesse et al., 2018; Pogue et al., 2018). Due to their high complexity and low frequency, important gaps still exist in their prevention, diagnosis, and treatment. Most of the patients with rare diseases receive treatments intended to alleviate the disease-derived complications and improve their quality of life, but without tackling the underlying disease cause. Indeed, for most rare pathologies, including the neurodegenerative ones, there is no treatment to prevent, delay, or cure the disease (Kaufmann et al., 2018), in particular in children (Raïs Ali and Tubeuf, 2019). There is then an urgent necessity to find therapeutic opportunities to fulfill these unmet needs. Recently, gene therapies were authorized for the treatment of neurodegenerative disorders. This is the case of onasemnogene abeparvovec (Zolgensma^®^), which, if administered early in life seems to cure spinal muscular atrophy (Hoy, 2019). However, these technologies, even if very promising, might be very costly and time-consuming in some cases. Because of that, leading pharmaceutical companies show relatively low interest in orphan drug research and development due to the high investment compared to the low market return that may get from the developed product (Pogue et al., 2018). According to the EU, 10% of orphan drug designations have a neurological indication (Morel et al., 2016). Drug repurposing–based approaches appear then as cost- and time-saving strategies for the development of therapeutic opportunities for rare diseases (Pushpakom et al., 2019). In this article, we discuss the scientific, regulatory, and economic aspects of the repurposed drugs proposed for the treatment of rare neurodegenerative disorders using as example the approaches taken for the treatment of Huntington’s disease, Friedreich’s ataxia, Wolfram syndrome, and amyotrophic lateral sclerosis, four rare neurodegenerative disorders (Matilla-Duenas et al., 2017). The role of academia, pharmaceutical companies, patient associations, and foundations in the identification of candidate compounds and their preclinical and clinical evaluation is also discussed.

### Drug Repurposing

Drug repurposing or repositioning implies the use of approved drugs or previously evaluated but unapproved active pharmaceutical compounds for the treatment of diseases or conditions different from their original medical indication (Pushpakom et al., 2019; Fetro and Scherman, 2020). In the past, this approach was only based on serendipitous findings in which a drug was found to have an additional on-target or off-target effect that could be eventually exploited for the treatment of other diseases (Ashburn and Thor, 2004). In recent years, the identification of repurposed drug candidates is based on systematic, computational, and/or experimental (patient-centered or drug-centered) approaches based on understanding the underlying pathophysiological disease mechanisms or having a better knowledge on the mechanism of the action of a given drug (Park, 2019; Pushpakom et al., 2019). The different strategies currently used for the identification of new therapeutic indications for existing drugs have been extensively reviewed by Pushpakom et al., 2019.

Compared to “*de novo*” drug discovery, drug repurposing constitutes a very attractive option to save time and money and reduce the risk of failure. Indeed, while some estimates find that the development of new drugs might take around 13–15 years with an overall investment of around US$ 1.3–3 billion to bring one drug to the market, it has been proposed that drug repurposing might allow to save 5–7 years, reduce the cost to around US$ 300 million per molecule, and the risk of failure from more than 95% for newly-designed drugs to around 45% for a repurposed one (Ashburn and Thor, 2004; Nosengo, 2016; Cha et al., 2018; Wouters et al., 2020). This is because the repurposed drugs have already passed preclinical testing, safety, and pharmacokinetic profiles from early-stage clinical trials, and often their formulation has already been developed (Ashburn and Thor, 2004; Nosengo, 2016; Cha et al., 2018). However, if the route of administration is changed, early phase I clinical trials need to be performed. The regulatory and phase III costs, however, are about the same as for newly developed drugs (Pushpakom et al., 2019). Even if drug repurposing offers very good advantages for the treatment of rare diseases, this approach does not always succeed. Indeed, the risk of late-stage failure is analogous to the one-off newly developed drugs, and sometimes the need for drug reformulations may be as costly as that for *de novo* drug development (Pushpakom et al., 2019; Fetro and Scherman, 2020).

In many European countries, the “off-label” use of repurposed drugs for rare diseases is relatively common, mostly in pediatric patients. This practice has some negative points; one is related with the liability with respect to the administration of medicinal products, since the marketing authorization holder is responsible for the adverse effects arising from the approved indication but not in the case of off-label use. In addition, the off-label administration of repurposed drugs prevents a proper documentation of their effects and safety. Therefore, it is much more advantageous and safer if the “off-label use” occurs within a clinical protocol as part of the repurposing process that will allow safe patient monitoring and a systematic data collection and analysis. For a good overview about the benefits and risks of the off-label use of repurposed medicinal products along with potential solutions to tackle the last issue, we suggest reading the study by Verbaanderd et al. (2019). According to the EU, the repurposing approval is frequently provided through a much simpler process than an initial marketing authorization. Indeed, a marketing authorization holder can apply for a type II variation of its authorized product for the addition of a new therapeutic indication under the same marketing approval (Verbaanderd et al., 2019). However, this fast procedure is only possible if no changes to the pharmaceutical form, strength, or route of the administration of the medicinal product were made. If alterations in one or more of the latter points are introduced, the marketing authorization of the repurposed drug will be considered an extension of the initial marketing authorization (Verbaanderd et al., 2019). In this case, the approval procedure will be the same as the one for newly designed drugs, and the market access for the new indication will be granted only if regulatory evidence of quality, efficacy, and safety is proven, and if the national criteria for coverage/reimbursement and pricing are satisfied (Fetro and Scherman, 2020). Regulatory approval is normally a requirement for inclusion in clinical guidelines and for reimbursement.

Additionally, even if one may think that repurposed drugs are cheaper (Chong and Sullivan, 2007), several repurposed medicinal products authorized by the European Medicines Agency (EMA) as orphan drugs are, for one reason or another, much more expensive than the original product for which the market price covered the cost for research and development. One example is mexiletine. Its price skyrocketed after receiving the European marketing authorization for the treatment of myotonia in patients with non-dystrophic myotonic disorders by the EMA. Naturally, this is only one example, and it does not imply that all drugs tend to follow the same price evolution. In fact, as described in more detail in the section “*Incentives for R&D and new drug development for rare diseases*”, there are several examples of drugs with a much cheaper price.

### Collaborative Models: The Role of Academia, Industry, and Patient Organizations in Drug Repurposing for Rare Diseases

Academic research importantly contributes to the first steps of the discovery of “*de novo*” or repurposed drugs by elucidating underlying disease mechanisms and identifying therapeutic targets (Stevens et al., 2011). Indeed, a report aiming to evaluate the role of academia on the identification and development of “*de novo*” or repurposed transformative drugs with groundbreaking effects on patient care pointed to a key role of academic medical centers, often funded by the government and/or by patient associations or foundations, in conceptualizing therapeutic approaches based on preclinical research disease mechanisms, and providing the proof of concept for the utilization of a given molecule for a particular disease (Kesselheim et al., 2015). This study also highlighted the importance of collaborations between academia and pharmaceutical industries to perform the follow-up steps of drug development to ensure further clinical testing and formulation of the newly discovered or repurposed drug (Kesselheim et al., 2015). Besides the individual efforts taken by academic institutions or pharmaceutical companies, public–private collaborative initiatives also exist to promote the discovery of new indications for existing drugs, for example, the Medical Research Council (MRC)–AstraZeneca compound collaboration in United Kingdom (https://mrc.ukri.org/funding/browse/mrc-az-cld/mrc-astrazeneca-centre-for-lead-discovery/mrc-az-centre-for-lead-discovery-cld-faqs/), and “Discovering New Therapeutic Uses for Existing Molecules (New therapeutic uses)” program from the National Center for Advancing Translational Sciences (NCATS) of the National Institutes of Health (NIH) in the United States (https://ncats.nih.gov/ntu/about). In both models, the MRC or the NIH provide funds to academic scientists to perform research in different disease areas, including rare disorders, and pharmaceutical companies such as AstraZeneca and NIH-industry partners grant access to their compound library, and their state-of-the-art high-throughput screening facilities (Rees et al., 2016). These public–private partnerships combining biological knowledge, financial support, and screening expertise contribute to accelerate the discovery of novel targets in a collaborative setting (Simpson and Reichman, 2014; Reichman and Simpson, 2016).

The Critical Path Institute (C-Path) is a non-profit, public-private partnership organization which has been working closely with experts from the pharmaceutical industry, academia, and the FDA in the context of collaborative approaches, where both sharing of data and expertise take place. Various programs are being conducted under the C-Path which include but are not limited to Critical Path to Therapeutics for Ataxia (CPTA), Huntington’s Disease Regulatory Science Consortium (HD-RSC), Critical Path for Alzheimer’s disease (CPAD), Critical Path for Parkinson’s (CPP), and Friedreich’s Ataxia-Integrated Clinical database (FA-ICD). In 2020, a public–private partnership dedicated to advance drug repurposing – CURE Drug Repurposing Collaboratory (CDRC, https://c-path.org/programs/cdrc) has been initiated by the C-Path and the FDA in collaboration with the NCATS. The goal of this collaborative initiative is to generate a platform where all the real-world clinical outcome data are open-sourced at one place and from which knowledge can be gained to enhance drug repurposing through the identification of lead candidates. Also, the platform will provide information about unmet medical needs for diseases, assistance in regulatory roadmaps, and during clinical trials to identify safe and effective drugs for new indications.

The importance of patient associations and advocacy groups in the development of therapeutic approaches has been recognized in the last years in all disease areas, including rare diseases. These organizations are now considered an integral part in the research process, since they foster collaborations between academia, pharmaceutical companies, and clinicians and act as a link between the patients and the researchers providing useful information about patient’s expectations and needs. They are also actively involved in shaping Consortia’s research agendas and help ensure the feasibility and success of research protocols by assisting with study design and patient recruitment. In addition, besides organizing educational programs, facilitating networking amongst patient groups, and providing patient services, they also raise funds to finance academic research preclinical projects and clinical trials (Merkel et al., 2016). One example is the International Rare Diseases Research Consortium (IRDiRC) which ‘unites national and international governmental and non-profit funding bodies, companies (including pharmaceutical and biotech enterprises), umbrella patient advocacy organizations, and scientific researchers to promote international collaboration and advance rare diseases research worldwide’ (https://irdirc.org/about-us/).

Most individual patient associations or foundations are, in general, part of bigger non-profit patient organizations such as the National Organization for Rare Disorders (Nord) (https://rarediseases.org/), EURORDIS (European Organization for Rare diseases, https://www.eurordis.org) or Findacure https://www.findacure.org.uk/. The Orphanet database (https://www.orpha.net/consor/cgi-bin/index.php) provides compiled information about rare diseases and patients’ organizations registered in Europe.

Concerning rare neurodegenerative diseases, the European Reference Network for Rare Neurological Disorders (ERN-RND) http://www.ern-rnd.eu/, established by the EU supports patients and families affected by rare neurological diseases and facilitates the participation of patients in clinical trials with repurposed medicinal products. The neurodegenerative rare diseases covered by this network include several ataxias and Huntington’s disease.

Cures Within Reach (https://www.cureswithinreach.org) is another philanthropic organization dedicated to fund research projects related with drug, device, and nutraceutical repurposing to provide fast and safe treatments for unmet clinical needs in different common and rare diseases. Healx, the only commercial company with this type of model (https://healx.io/), combines artificial intelligence and collaborates with academic institutions, biotech, pharma, and patient groups to identify and progress novel therapies. Currently, Healx has 18 therapies listed in the pipeline.

### Incentives for R&D and New Drug Development for Rare Diseases

Understanding the drivers of pharmaceutical research and development (R&D) is important to foster innovation in the pharmaceutical market. The pharmaceutical industry is responsive to the potential market size: when it increases, the entry of new non-generic drugs and new molecular entities (i.e., those more profitable) also increases (Acemoglu and Linn, 2004). This constitutes an issue in the case of rare diseases. In fact, the interest of pharmaceutical companies in orphan drug development is traditionally low due to the relatively high cost of investment compared to the low market return of the product, precisely because of the small market size (https://www.eurordis.org/IMG/pdf/princeps document-EN.pdf). Given the lack of competition, therapy products for rare diseases have a commercial potential, namely, when their market price is extremely high. For instance, gene therapy onasemnogene abeparvovec (Zolgensma^®^) has a market price of more than €2m. The justification for setting a high market price is the high costs of R&D, manufacturing and distribution, and the small market size. But by setting such a high price, potentially lifesaving therapies are prohibitive for most patients and, in practice, patients cannot have access to these medicinal products (Fischer et al., 2019).

Nevertheless, the price of drugs to treat rare diseases varies considerably. If we consider the case of repurposed drugs for Huntington’s disease currently available in Belgium, the average price is €1,280, with the cheapest drug listing a public price of €11,09 and the most expensive one listing a price equal to €4,982.

Verifying the veracity of high development costs is generally difficult. As a matter of fact, it is challenging to estimate the costs of drug development, namely, because pharmaceutical companies do not generally make the costs of drug development publicly available. Nevertheless, Dimasi et al., 2016 found that it can be more than $2b, while Wouters et al., (2020) estimated a mean of approximately $1.3b, with the latter including a large sample of orphan drugs. Jayasundara et al., (2019) found a higher clinical cost of drug development for non-orphan drugs with respect to orphan drugs. Despite the evidence of lower costs for developing orphan drugs, there is a lack of drugs to treat rare diseases.

Additionally, pharmaceutical companies might take into consideration potential competition from drugs previously adopted in the market with the same active ingredient but approved for other authorized indication or patient groups. In this situation, the drug might have a lower price or can even face competition from a generic drug. Therefore, companies could be discouraged from trying to repurpose the drug as they would pay for the research and development, while the company which has the older product in the market would take the profits. In order to quantify the extent to which this situation impacts the decision of pharmaceutical companies to try to repurpose drugs, one would need to know how many of the repurposed drugs actually have a generic competitor, for how long, and how many patients would be treated by the older drug in order to estimate potential foregone profits. In the examples considered in this article, this is almost never the case. Due to the recognition of a need to foster the development of medicinal products to treat rare diseases, different economic and regulatory incentives have been provided to the sponsors of orphan products worldwide. The first specific regulation, the Orphan Drug Act (ODA), was approved by the US in 1983, and its incentives include 1) 7 years of market exclusivity to sponsors of approved orphan products; 2) tax credits; and 3) research grants. In Europe, the regulation on orphan medicinal products (Regulation (EC) No 141/2000) provides different incentives to sponsors, such as a) scientific advice on study protocols provided by the EMA (protocol assistance); b) 10 years of market exclusivity, which can be extended by 2 years for pediatric investigation; and c) reduced fees for regulatory activities. These incentives have attracted small and medium enterprises (SMEs), academia, pharmaceutical companies, public–private partnerships, and patient advocacy groups to work in rare diseases.

In spite of these incentives, according to the EU in 2020, there were over 2,380 medicines with orphan designation but, as of August 2021, only a few more than 200 drugs had marketing authorization (https://www.ema.europa.eu/en/documents/report/annual-report-use-special-contribution-orphan-medicinal-products-2020_en.pdf). Therefore, rare diseases are still underserved in terms of drug development in comparison with non-rare diseases. Considering the need of addressing unmet needs of rare diseases’ patients, the European Commission launched an initiative which aims at revising the legislation to incentivize the development of medicines for rare diseases and children (Commission, 2021). The objective is to provide solutions to possible shortcomings of the current legislation and take into account the exclusive role of member states in crucial areas such as pricing and reimbursement of medicines. This will also be addressed within the Pharmaceutical Strategy for Europe, which has a broader set of objectives, including fostering healthcare and pharmaceutical innovation (Commission, 2020). The low number of orphan drugs authorized with respect to the large number of orphan designations is explained by the high investment needed for new drug development, and the long duration of the process that is accompanied by the regulatory hurdles and organizational issues faced while performing clinical trials in rare diseases (Ashburn and Thor, 2004; Wastfelt et al., 2006). Indeed, despite the availability of original safety data on the previous approved indication, to receive marketing authorization, the repurposed drugs need to be tested for their efficacy, safety, and tolerability in clinical trials. These clinical studies conducted to provide the benefit/risk data are expensive and complex due to the frequent complexity of the diseases, the low number of patients affected, and their wide geographical distribution (Ashburn and Thor, 2004; Wastfelt et al., 2006). Additionally, within rare diseases, pharmaceutical companies tend to target more profitable areas. In fact, R&D is higher for diseases with an older average age at death (i.e., in adulthood instead of infancy or childhood), which provides additional evidence that R&D is concentrated in more profitable areas; and in rare diseases in high-prevalence categories, which corroborates evidence that market share is a driver of R&D (Raïs Ali and Tubeuf, 2019).

### Business Models for Drug Repurposing

Traditionally, the business model of leading pharmaceutical companies consisted in in-house drug development, from R&D until commercialization, and a general focus on blockbuster drugs. However, this is changing for several reasons, such as rising development costs, competition from generics, and end of patents of some blockbuster drugs (Phillips, 2013). The industry is facing an increasing productivity gap because the cost per new drug is growing while the number of new drug introductions is not accompanying this increase (Grabowski, 2004).

Drug repurposing, namely, for rare diseases can be an interesting business for pharmaceutical companies and has been considered a possible response to the productivity gap (Boguski et al., 2009). In the last 20–25 years, a number of companies and non-profit organizations devoted to drug repurposing have emerged, with a reduced number of failures (Naylor et al., 2015). Meanwhile, some of these companies were acquired by larger ones, and some leading pharmaceutical companies have created departments devoted to repurposing (Sleigh S. H. and Barton C. L., 2010). More recently, several drugs repositioned for COVID-19 are being considered, which has increased the interest in drug repurposing.

As mentioned before, from a business perspective, drug repurposing in rare diseases can be attractive, namely, because of its reduced costs compared to *de novo* drug development, potentially diminished risk of failure, reduced time required for approval, and higher pricing. However, a significant proportion of rare diseases affect children, which represents a challenge since in most cases the clinical trials conducted for the drugs to be repurposed only included adults. Despite drug repurposing being a viable strategy to find treatments for some rare diseases, new business models are needed to foster this approach. This includes collaborative strategies combining the strength of different agents, such as pharmaceutical and biotechnology companies, venture capitalists, and academia (Pushpakom et al., 2019). Cha et al. (2018) identified three key players in the market, each one with a different business model: academia/research institutes, repurposing technology companies, and pharmaceutical companies. The former, that usually sponsors a significant proportion of phase I and phase II trials for repurposed drugs, faces lower economic or commercial constraints while being more dependent on public funding. The repurposing technology companies are bound by their business model, which includes consulting services, offering drugs databases, and drug pipelines (Sleigh SH. and Barton CL., 2010; Sleigh S. H. and Barton C. L., 2010; Naylor et al., 2015). Some of these companies collaborate with leading pharmaceutical companies. Pharmaceutical companies have a more prominent role in drug discovery and development and tend to be profit seekers. A few might have some ethical concerns or embrace, for example, corporate social responsibility. Overall, there is no unique or widely adopted business model used by pharmaceutical companies for drug repurposing, but three business models frequently used have been identified (Naylor et al., 2015): the in-house model, in which the pharmaceutical companies have their own department or resources devoted to drug repurposing (this model has already been abandoned by some companies); the out-licensing model, in which pharmaceutical companies provide access to their compounds on an out-licensing basis (this limits exposure to risk and additional costs for the corresponding compound); and the extended profiling model, in which a drug candidate starts being evaluated for new indications immediately after a successful first-in-human study.

## Drug Repurposing for Rare Neurodegenerative Diseases

### Huntington’s Disease

#### Clinical Features and Genetic Cause

**FIGURE 1:**
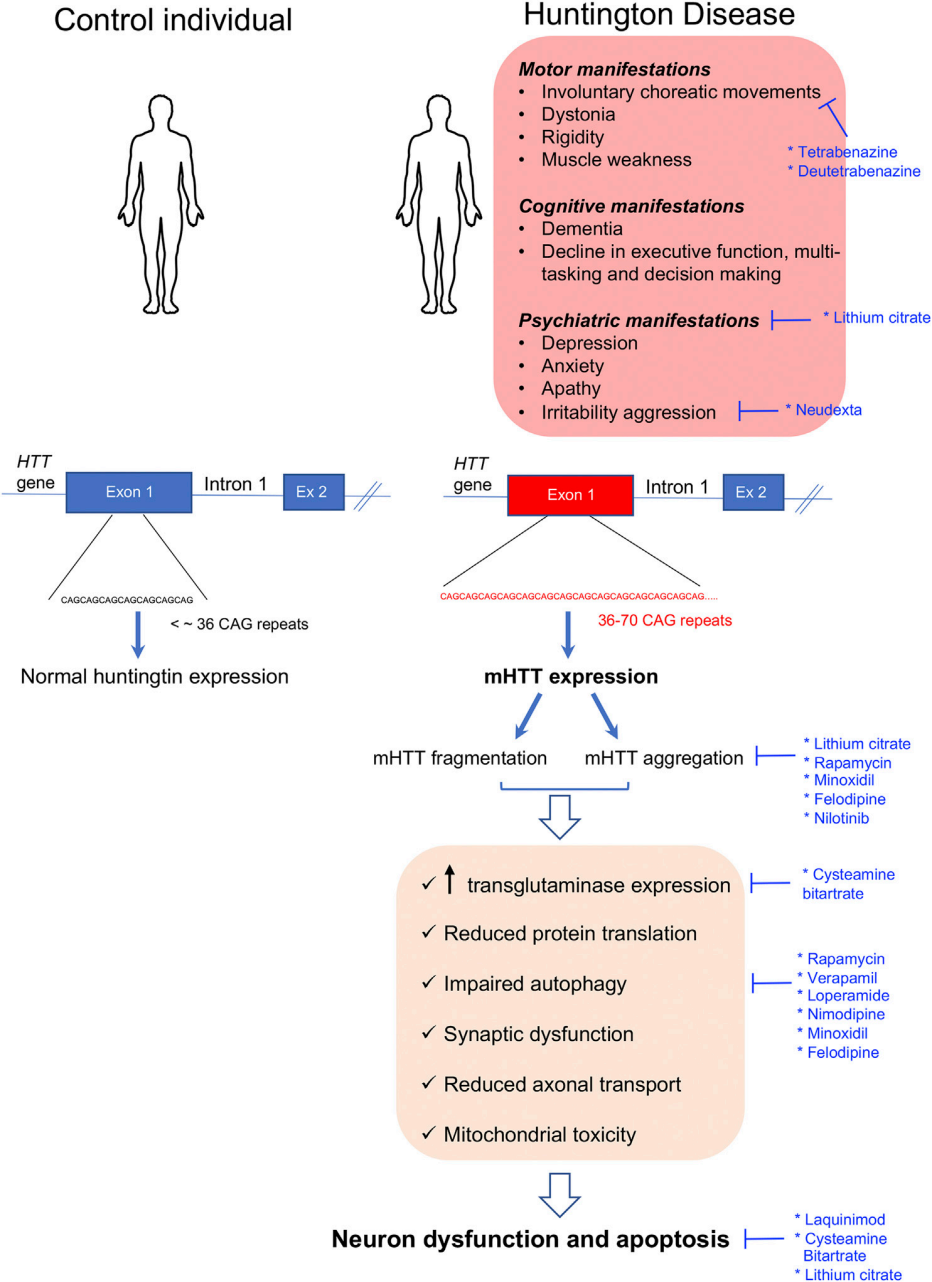
Clinical manifestations and molecular disease mechanisms in Huntington’s disease. This pathology is caused by CAG repeat expansions in the first exon of the HTT gene which result in the accumulation of mutant Huntington protein (mHTT) within the cells which causes a wide variety of cellular alterations leading to neuronal dysfunction and death. Repurposed drugs with and without orphan designation for Huntington’s disease are highlighted in blue along with the cellular dysfunctions or clinical manifestations that they tackle. Ex: exon.

Huntington’s disease (HD) is the most common rare neurodegenerative disorder with an estimated prevalence of 1/20,000 to 1/10,000 in the Caucasian population. It was discovered by George Huntington in 1872. The disease is characterized by a triad of motor, cognitive, and psychiatric manifestations (Ghosh and Tabrizi, 2018). Motor features include involuntary choreatic movements, dystonia, and rigidity, while the behavioral and psychiatric disorders include depression, anxiety, apathy, irritability aggression, and dementia among others. The clinical manifestations usually appear during the third decade of life and become fatal after 15–20 years due to progressive neuronal dysfunction and ultimate neuronal death (Ghosh and Tabrizi, 2018). The diagnosis of this disease is usually performed by molecular genetic testing followed by computerized tomography scanning, magnetic resonance imaging (MRI), and electroencephalography. HD is an autosomal dominant disorder caused by an unstable 36–70 CAG trinucleotide expansion in exon 1 on the Huntington gene (*HTT*) (Snell et al., 1993) (Figure 1). The onset and the severity of the disease is dependent on the length of the CAG repeat, with longer repeats being associated with more severe phenotypes (Nance, 2017; Caron et al., 2018).

### Molecular Phenotype

The *HTT* gene encodes for a large 350-kDa protein, also called HTT, which is ubiquitously expressed but enriched in the brain (Aronin et al., 1995; Trottier et al., 1995). The normal function of HTT is currently unknown. HTT is folded in a super helical structure containing a hydrophobic core. In the mutated HTT (mHTT), the CAG expansion results in a polyglutamine (polyQ) tract starting in residue 18 of the polypeptide. This expanded polyQ region may be proteolytically cleaved, and it has been proposed that the mHTT fragments generated may induce neurodegeneration (Halliday et al., 1998; Barbaro et al., 2015; Ghosh and Tabrizi, 2018). In addition, it was shown that the mHTT protein is highly aggregation-prone, resulting in intranuclear and intracytoplasmic inclusions throughout the brain (Difiglia et al., 1997). Many studies report that mHTT aggregates can be either toxic or protective depending on the disease stage, their subcellular localization, and their association with other partners or organelles (Saudou et al., 1998; Slow et al., 2003; Slow et al., 2005; Leitman et al., 2013). When present in the nucleus, these aggregates sequester transcription factors resulting in gene expression alterations (Labbadia and Morimoto, 2013), while when present in the cytosol, they may bind to diverse proteins resulting in an altered autophagy-lysosome pathway, reduced protein translation, synaptic dysfunction, reduced axonal transport, mitochondrial toxicity, and energy imbalance among others (Labbadia and Morimoto, 2013; McColgan and Tabrizi, 2018) (Figure 1). There is currently no treatment to prevent or delay the progression of HD; however, some pharmaceutical and non-pharmaceutical interventions (physiotherapists, psychologists, and social workers) aiming to relieve the multiple symptomatic manifestations of the disease are beneficial for some patients and contribute to improve their quality of life (Frank, 2014). Within the pharmaceutical approaches, some neuroleptics with anti-dopaminergic activity or acting as selective serotonin reuptake inhibitors (SSRIs) may be used in the management of psychosis-associated symptoms in HD-like anxiety, depression, and irritability (Mittal and Eddy, 2013; Unti et al., 2017).

### Approved and Non-Approved Orphan-Designated Repurposed Drugs for Huntington’s Disease

Tetrabenazine (Xenazine^®^), originally indicated as an anti-psychotic drug, is a repurposed molecule approved in 2008 by the U.S. Food and Drug Administration (FDA) for the treatment of chorea in HD (Jankovic and Clarence-Smith, 2011). This authorization was based on the positive results of four controlled clinical trials performed in the United States with HD patients (Huntington Study, 2006; Frank et al., 2008; Frank, 2009). Tetrabenazine is a reversible vesicular monoamine transporter 2 (VMAT2) inhibitor which blocks the uptake of cytosolic monoamines and prevents dopamine release from synaptic vesicles (Login et al., 1982). An extensive review on the mechanism of action, pharmacodynamics, pharmacokinetics, and metabolism of this drug can be found in the study by Jankovic and Clarence-Smith, (2011). Following the positive results from a randomized double-blind, placebo-controlled trial, in April 2017, the FDA also approved AUSTEDO (deutetrabenazine), another molecule with VMAT2 inhibitor activity, to treat chorea in HD (Huntington Study et al., 2016).

Some repurposed medicinal products have received the orphan designation by the EMA for the treatment of Huntington’s disease but have not been authorized yet: for example, cysteamine bitartrate (https://www.ema.europa.eu/en/medicines/human/orphan-designations/eu3141306) and lithium citrate tetrahydrate (https://www.ema.europa.eu/en/medicines/human/orphan-designations/eu309706). In the EU, cysteamine bitartrate is used under the name of Cystagon^®^ and Procysbi^®^ for the treatment of nephropathic cystinosis. In HD, this drug was shown to be neuroprotective in several mouse models by improving weight loss and motor abnormalities and prolonging animal survival. It has been proposed that the drug may reduce nerve damage and improve motor function by blocking the activity of the enzyme transglutaminase, shown to be increased in HD patients and involved in nerve injury, by increasing the secretion of the brain-derived neurotrophic factor (BDNF) that improves neuron survival and function, and by other still unidentified mechanisms (Dedeoglu et al., 2002; Karpuj et al., 2002; Van Raamsdonk et al., 2005; Bailey and Johnson, 2006; Borrell-Pages et al., 2006; Arbez et al., 2019). A randomized, double-blind, placebo-controlled trial with cysteamine bitartrate showed that the drug is safe and well tolerated by HD patients, but failed to demonstrate efficacy in the full-patient cohort (Verny et al., 2017). A post hoc analysis in which patients were stratified by disease severity based on their initial motor scores suggested that the drug reduced the progression of the disease in patients with the most severe motor impairment. Further clinical studies are needed to prove the efficacy of the drug. Lithium is an inhibitor of glycogen synthase kinase-3 and inositol monophosphatase that have been used as mood stabilizers for several decades (Yatham et al., 2018). Recent studies performed in several preclinical HD models suggest that this molecule is able to increase the clearance of intracellular protein aggregates, to confer neuroprotection, and to improve motor dysfunction and coordination (Scheuing et al., 2014). Several blind and unblind clinical studies using lithium for short periods of time in HD patients showed improvement in choreatic movements, motor function, and mood stabilization in some but not all patients (Anden et al., 1973; Dalen, 1973; Mattsson, 1973; Aminoff and Marshall, 1974; Danivas et al., 2013; Raja et al., 2013). Further blinded trials with larger patient cohorts are needed to determine the effectiveness of this drug. Table 1 provides further information about the aforementioned drugs, including pharmaceutical companies or academic institutions involved in designing and running the clinical trials, whether the drugs received orphan designation from the EMA and/or the FDA, and the sponsors that made the orphan designation request.

**TABLE 1:** List of the repurposed drugs, with and without orphan designation or drug marketing authorization for Huntington’s disease (HD) mentioned in this article. The sponsors (entities involved in making the orphan designation request to the EMA or the FDA), and the public or private organizations involved in designing and running the clinical trials are detailed. N/A = non-applicable.

Drug name	Orphan drug designation for HD?	Sponsor	Information about the sponsors	Drug marketing authorization for HD?	Original indication of the drug	Mechanism of action and preclinical findings	Outcome of the clinical trials	Entities involved In designing and running the clinical trials	References
Tetrabenazine (Xenazine^®^)	Yes: FDA, 1997	*US:* Prestwick Pharmaceutical, Inc., US	Pharmaceutical company focused on chronic CNS diseases	Yes	Anti-psychotic	Is a vesicular monoamine transporter 2 inhibitor. It blocks the uptake of cytosolic monoamines and prevents dopamine release from synaptic vesicles	The drug was safe and well tolerated in clinical trials. Positive results were obtained from four controlled clinical trials performed in the US.	Trials sponsored by the Huntington’s Study Group, or the University of Rochester, US, or the Assistance Publique - Hôpitaux de Paris, or the Ohio State University, United States, or Prestwick Pharmaceuticals or Boston University, MA, US; with the collaboration of Lundbeck LLC, and academic institutions and hospitals in the US	Huntington Study, (2006); Frank et al. (2008); Frank. (2009); Jankovic and Clarence-Smith. (2011) https://clinicaltrials.gov/ct2/show/NCT01451463?cond=Huntington+Disease&draw=2&rank=60 https://clinicaltrials.gov/ct2/show/NCT00632645?cond=Huntington+Disease&draw=2&rank=100
Cysteamine bitartrate (Cystagon^®^ and Procysbi^®^)	Yes: EMA, 2014; FDA, 2008	*Europe:* Raptor Pharmaceuticals Europe BV, the Netherlands *, the orphan designation was then transferred to Chiesi Farmaceutici S.p.A., Italy §. US: Horizon Therapeutics United States, Inc. &	*European pharmaceutical company. § Leading international pharmaceutical company, and certified B Corporation. & International pharmaceutical company focused on for rare, autoimmune, and severe inflammatory diseases	No	Nephropathic cystinosis	Transglutaminase inhibition, enhancement of BDNF levels, and additional neuroprotective pathways to be determined. In rodent models of HD, the drug conferred neuroprotection and increased survival	The drug was safe and well tolerated in a randomized, double-blinded, placebo-controlled trial with HD patients but failed to demonstrate efficacy in the full patient cohort. A post hoc stratified analysis suggested that the drug may reduce disease progression in patients with severe motor impairment	Various academic institutions in France	Dedeoglu et al. (2002); Karpuj et al. (2002); Van Raamsdonk et al. (2005); Bailey and Johnson. (2006); Borrell-Pages et al. (2006); Verny et al. (2017); Arbez et al. (2019)
Lithium citrate	Yes: EMA, 2010; FDA, 2010	*Europe and US:* Medesis Pharma, SA, France	Pharmaceutical Biotech company	No	Mood stabilizer	Inhibitor of glycogen synthase kinase-3 and inositol monophosphatase. In HD preclinical models, the drug improved motor phenotype through a still unknown mechanism	Several uncontrolled studies showed beneficial motor and psychiatric effects. Further trials are needed	Academic institutions	Anden et al. (1973); Dalen. (1973); Mattsson. (1973); Aminoff and Marshall. (1974); Danivas et al. (2013); Raja et al. (2013)
Laquinimod	Yes: FDA, 2017	Active Biotech AB, Sweden	Biopharmaceutical company	No	Immunomodulatory drug used in multiple sclerosis	Immunomodulatory drug. The drug reduces apoptosis and improves motor and psychiatric phenotypes in mouse models of HD	A 1-year phase II clinical trial in HD showed no effects on the motor score but a significant reduction in caudate atrophy in patients with early HD.	Trial sponsored by Teva Branded Pharmaceutical Products R&D, Inc	Garcia-Miralles et al. (2016); Caron et al. (2018) https://clinicaltrials.gov/ct2/show/NCT02215616?cond=Huntington+Disease&draw=2&rank=87
Rapamycin (sirolimus) Rapamune^®^	No	N/A	N/A	No	Indicated to prevent organ transplant rejection	Immunosuppressor. In HD preclinical models the drug induced autophagy and cleared polyglutamine aggregates reducing their toxicity	No clinical trials available	N/A	Ravikumar et al. (2002); Ravikumar et al. (2004); Sarkar et al. (2005); Sarkar et al. (2007a); Sarkar et al. (2007b)
Minoxidil	No	N/A	N/A	No	Antihypertensive and vasodilator used in case of severe hypertension. Also used to prevent hair loss	Autophagy inducer. The drug cleared mHTT aggregation in fly and zebrafish models of HD.	No clinical trials available	N/A	Williams et al. (2008)
Felodipine Plendil^®^	No	N/A	N/A	No	Antihypertensive	Autophagy inducer. In mouse models of HD, the drug clears mHTT	No clinical trials available	N/A	Siddiqi et al. (2019)
Nilotinib (Tasigna^®^)	No	N/A	N/A	No	Anticancer drug in chronic myeloid leukemia	Tyrosine kinase inhibitor. The drug enhanced the clearance of mHTT and showed protective brain effects in preclinical studies	Phase Ib clinical trial is ongoing	Trial sponsored by Georgetown University, US in collaboration with Cures Within Reach (patient association)	Pagan et al. (2016); Pagan et al. (2020); Pagan et al. (2021) https://clinicaltrials.gov/ct2/show/NCT03764215?cond=Huntington+Disease&draw=2&rank=18
Dextromethorphan/quinidine (Neudexta^®^)	No	N/A	N/A	No	Treatment of pseudobulbar effect (condition of contextually inappropriate/exaggerated emotional expression)	Drug proposed for the treatment of irritability in HD.	A randomized, crossover quadruple-blind clinical trial with 22 HD patients to evaluate the safety and tolerability of the drug was initiated in 2019	Trial sponsored by the University of Texas Health Science Center, Houston, US in collaboration with. Cures Within Reach (patient association)	Cummings et al. (2015). https://clinicaltrials.gov/ct2/show/NCT03854019

The following websites were consulted to build up this table. Community Register of orphan medicinal products from the European commission: https://ec.europa.eu/health/documents/community-register/html/reg_od_act.htm?sort=a; Orphan Drug Designations and Approvals from the. FDA: https://www.accessdata.fda.gov/scripts/opdlisting/oopd/.ClinicalTrial.gov to retrieve the list of clinical trials for HD: https://clinicaltrials.gov/ct2/results?cond=Huntington+Disease&Search=Apply&age_v=&gndr=&type=&rslt=.

Additional new and repurposed drugs that received orphan designation for HD but are not yet approved for its treatment can be found in the Orphanet portal (https://www.orpha.net/consor/cgi-bin/index.php).

### Additional Drug Repurposing–Based Therapeutic Strategies Under Investigation (Non-Orphan Designated Drugs)

Without being an exhaustive list, here we will provide some examples of current drug repurposing–based approaches for HD at different stages of development (Table 1). A detailed review about all ongoing “*de novo*” or repurposing-based strategies for HD treatment can be found in the study by Caron et al., (2018).

As mentioned above, mHTT aggregates may compromise autophagic clearance by perturbing cargo recognition and autophagosome motility which results in cell death (Martinez-Vicente et al., 2010; Zatyka et al., 2020). In fly and mouse models of HD, rapamycin-mediated mTOR inhibition enhanced the autophagic flux and clearance of unfolded mHTT resulting in reduced toxicity (Ravikumar et al., 2002; Ravikumar et al., 2004; Sarkar et al., 2005; Sarkar et al., 2007a; Sarkar et al., 2007b). Since rapamycin has numerous side effects, Williams et al. (2008) screened a library of FDA-approved drugs looking for autophagic enhancers with mTOR-independent activity. This screening identified clonidine, verapamil, loperamide, nimodipine, and minoxidil among others as drugs with autophagic enhancing activity. *In vitro* assays showed that all of them were able to reduce mHTT aggregation and toxicity in neuroblastoma cells (Williams et al., 2008). The authors also showed that calpain inhibition reduced mHTT aggregation and toxicity. Similarly, the antihypertensive drug (L-type calcium channel blocker) felodipine was shown to induce autophagy and clear mHTT in a mouse model of HD (Siddiqi et al., 2019).

Abnormal immune activation and inflammatory processes resulting from the central nervous system (CNS) and peripheral immune cell dysfunction have also been highlighted as important contributors to the pathophysiology of HD (Bjorkqvist et al., 2008). Laquinimod is an immunomodulatory drug developed for the treatment of multiple sclerosis. In the context of HD, it was shown that *in vitro*, this molecule reduces apoptosis in primary neurons derived from YAC128 mice, a HD model (Garcia-Miralles et al., 2016). Moreover, chronic laquinimod administration in these animals improved white matter integrity, motor and psychiatric phenotypes, and reduced IL-6 serum levels (Garcia-Miralles et al., 2016). A 12-month phase II clinical trial for HD with this molecule showed no effect on the motor score but revealed a significant reduction in caudate atrophy that was more evident in patients with early HD (Caron et al., 2018), pointing to a promising role of immunomodulators for the treatment of HD. Further clinical studies are required to support the neuroprotective effect of all these drugs in HD patients.

Cures Within Reach is currently funding two clinical trials in HD with repurposed drugs. One of them, led by Dr. Anderson from the Georgetown University, will study the safety and tolerability of nilotinib, a FDA-approved cancer drug for the treatment of chronic myeloid leukemia, in 10 HD patients with early-to-moderate HD. Based on previous findings from clinical trials with nilotinib in Parkinson’s disease (Pagan et al., 2016; Pagan et al., 2020; Pagan et al., 2021) they hypothesize that this drug may contribute to reduce the accumulation of toxic mHTT and have protective brain effects in HD. The second trial is led by Dr. Furr-Stimming from the University of Texas and aims to study the safety and tolerability of Neudexta, a drug currently used for the treatment of the pseudobulbar effect. This drug was shown to importantly ameliorate agitation in patients with Alzheimer’s disease (Cummings et al., 2015). Based on that, it was hypothesized that it may be useful to treat irritability in HD.

### Huntington’s Disease Patient Associations and Foundations

The International Huntington’s Disease Association http://huntington-disease.org/is a multinational federation created in 1979 that resembles 32 different Huntington’s disease societies from all over the world. The member societies promote medical professional education; provide individual and family support; and fund psychosocial, clinical, and biomedical research related with Huntington’s disease in their respective countries.

In addition, the Huntington’s disease coalition for the patient engagement (HD-COPE) is a global Huntington’s disease patient advocacy organization working in collaboration with the European Huntington’s Association (EHA), Huntington’s Disease Society of America (HDSA), and Huntington’s Society of Canada (HSC) formed in September 2017 to give patients’ voice in the clinical trials (https://hdsa.org/news/global-huntingtons-disease-patient-advocacy-organizations-unite-to-form-huntingtons-disease-coalition-for-patient-engagement-hd-cope/).

### Friedreich’s Ataxia

#### Clinical Features and Genetic Cause

**FIGURE 2:**
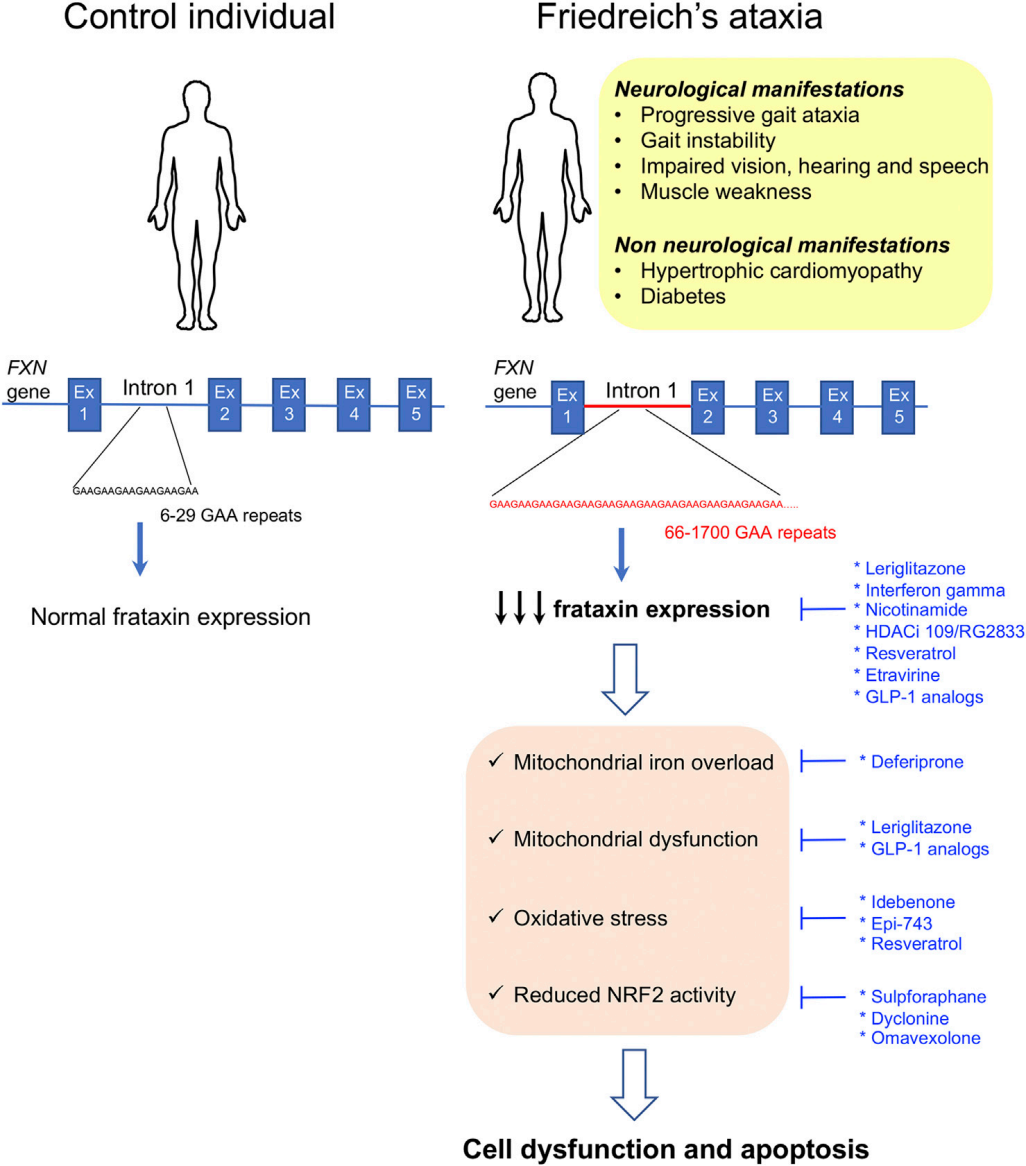
Clinical manifestations and molecular disease mechanisms in Friedreich’s ataxia. This pathology is caused by GAA repeat expansions in the first intron of the FXN gene. This results in reduced frataxin protein expression which causes a wide variety of cellular alterations leading to cell dysfunction and death. Repurposed drugs with and without orphan designation for Friedreich’s ataxia are highlighted in blue along with the cellular dysfunctions or clinical manifestations that they tackle. Ex: exon.

Friedreich’s ataxia (FRDA) is an autosomal recessive rare neurodegenerative disease mainly present within Caucasians. In this population, its prevalence ranges from 1/20,000 to 1/50,000, but large regional differences have been reported in Europe (Campuzano et al., 1996; Vankan, 2013). The disease is rare in sub-Saharan populations and very rare in the Far East (Vankan, 2013). The clinical manifestations include progressive limb incoordination (ataxia), gait instability, impaired vision, hearing and speech, and scoliosis and muscle weakness as a consequence of the progressive degeneration of the dorsal root ganglia neurons followed by neuronal loss in the cerebellar dentate nucleus and spinocerebellar tract degeneration (Koeppen et al., 2007; Marmolino, 2011; Koeppen et al., 2016; Selvadurai et al., 2018). In addition, as the disease progresses, non-neurological features appear, such as hypertrophic cardiomyopathy, that is the underlying cause for premature death (Harding, 1981; Pandolfo, 2009; Raman et al., 2011; Payne and Wagner, 2012; Parkinson et al., 2013), and diabetes that occurs in 30% of the patients as a result of increased pancreatic β-cell dysfunction and death in the context of insulin resistance (Finocchiaro et al., 1988; Schoenle et al., 1989; Cnop et al., 2012; Cnop et al., 2013; Igoillo-Esteve et al., 2015). In 96% of the patients, the disease is caused by homozygous trinucleotide GAA repeat expansions (from 70 to around 1700 triplets) in the first intron of the frataxin gene (*FXN),* while the remaining 4% have *FXN* point mutations (Campuzano et al., 1996; McCormack et al., 2000). The GAA repeat expansions interfere with transcription by heterochromatin silencing (Campuzano et al., 1996; Saveliev et al., 2003; Herman et al., 2006; Silva et al., 2015). The length of the GAA repeat is not identical in both alleles, and the allele with the shortest expansion size determines the residual *FXN* levels. Longer GAA repeats result in a more severe reduction in *FXN* expression (65–95% decrease with respect to healthy controls) and are associated with early disease onset and greater disease severity (Campuzano et al., 1996; Al-Mahdawi et al., 2008; Koeppen, 2011) (Figure 2).

### Molecular Phenotype

*FXN* encodes for a 210-amino acid mitochondrial protein called frataxin that regulates iron homeostasis by modulating iron storage, iron–sulfur cluster (Fe–S), and heme biosynthesis and iron carriage (Adamec et al., 2000; Emond et al., 2000; Schulz et al., 2000; Adinolfi et al., 2002; Becker et al., 2002; Gerber et al., 2003; Yoon and Cowan, 2003; 2004; Adinolfi et al., 2009; Paupe et al., 2009; Condo et al., 2010; Tsai and Barondeau, 2010). Accordingly, it was shown that reduced frataxin expression results in impaired function and/or expression of FeS-containing enzymes, such as catalase, and several respiratory chain proteins resulting in iron accumulation in the mitochondrial matrix, mitochondrial dysfunction, and oxidative stress (Babcock et al., 1997; Rotig et al., 1997; Puccio et al., 2001). In several FRDA models, frataxin deficiency has been associated with reduced NRF2 (nuclear factor (erythroid-derived 2) 2–like transcription factor) levels (Paupe et al., 2009; D’oria et al., 2013; Shan et al., 2013). NRF2 exhibits antioxidant properties by regulating the expression of antioxidant and cytoprotective genes (Kim et al., 2001). It also reduces inflammation, improves mitochondrial function, and maintains protein homeostasis (Dinkova-Kostova et al., 2018). Therefore, reduced NRF2 expression or activity importantly contributes to frataxin deficiency–induced cytotoxicity in FRDA (Figure 2). There is currently no approved therapy to prevent, delay, or revert the manifestations of the disease; however, the beneficial effect of many molecules targeting different aspects of the FRDA pathophysiology has been tested or is currently under study. In the beginning, the therapeutic approaches for FRDA were mostly focused on treating the downstream effects of frataxin deficiency, namely, improving iron homeostasis and mitochondrial function or reducing oxidative stress. In recent years, many of the approaches under study are centered on the upregulation of frataxin protein expression to restore frataxin levels. Indeed, increasing frataxin protein expression in FRDA patients to the levels found in carrier individuals that are asymptomatic is expected to provide a cure for the disease and stabilize disease progression. Compiling expert information on previous and current research studies for the treatment of FRDA can be found in the study by Clay et al., (2019).

#### Approved and Non-Approved Orphan-Designated Repurposed Drugs for Friedreich Ataxia

As mentioned above, there are currently no approved drugs for the treatment of FRDA, but several repurposed medicinal products have received orphan designation for the treatment of this disease. Deferiprone (orphan-designated by the FDA) and idebenone, omaveloxolone, alpha-tocotrienol quinone, leriglitazone, and interferon gamma (orphan-designated by both the EMA and FDA) are only some examples (https://www.orpha.net/consor/cgi-bin/index.php).

Deferiprone is a permeable iron chelator that alleviates mitochondrial iron overload and is indicated for the treatment of sickle cell disease (Pandolfo and Hausmann, 2013). Based on this feature, it was proposed that it could be beneficial for FRDA individuals. A 6-month double-blind placebo-controlled study with this molecule in pediatric and adult FRDA patients showed that deferiprone is relatively safe at the lower doses tested (20 mg/kg/day) and contributes to reduce cardiac hypertrophy. However, patients receiving higher deferiprone doses (40–60 mg/kg/day) presented worsening in their ataxic phenotype (Pandolfo et al., 2014). In this trial, the lack of deterioration in the placebo group did not allow for detection of any potential protective effect of deferiprone on the neurological manifestations of the disease. However, a post hoc subgroup analysis suggested that 20 mg/kg/day deferiprone may reduce disease progression in patients with less severe disease symptoms, pointing to the need of further clinical trials with selected patient populations to confirm or rule this beneficial effect.

The potential therapeutic benefits of idebenone, an antioxidant drug initially developed by Takeda Pharmaceutical for the treatment of Alzheimer’s disease and other cognitive defects, and currently approved to treat visual impairment in adolescents and adults with Leber’s hereditary optic neuropathy (Lyseng-Williamson, 2016), have been largely studied in preclinical models of FRDA, where it reduced apoptosis and showed cardioprotective effects (Jauslin et al., 2002; Seznec et al., 2004). Initial clinical studies suggested that this drug may have some cardioprotective effects in FRDA patients (Rustin et al., 1999; Hausse et al., 2002; Mariotti et al., 2003), but follow-up trials showed no cardioprotection (Lagedrost et al., 2011; Cook et al., 2019). In addition, these and other clinical studies (Rustin et al., 1999; Hausse et al., 2002; Buyse et al., 2003; Mariotti et al., 2003; Rustin et al., 2004; Rinaldi et al., 2009; Lynch et al., 2010) showed no neuroprotective effects. Only one open-label trial showed some neurological improvement in pediatric FRDA patients treated with this drug (Meier et al., 2012). Despite the potential cardioprotective effects of the molecule, the lack of neuroprotective properties seen in most of the studies makes this drug less attractive for FRDA treatment.

As mentioned above, frataxin depletion may cause NRF2 inactivation contributing to cell death. Enhancement of NRF2 expression or activity is considered a potential therapeutic approach to prevent neurodegeneration in FRDA (Petrillo et al., 2017). In addition, it was recently found that the *FXN* gene contains three antioxidant-responsive element (ARE) sites in its promoter region (Sahdeo et al., 2014), suggesting that NRF2 may modulate frataxin expression by binding to these ARE elements. In line with that, several compounds with NRF2-inducing activity, such as sulforaphane, the anti-epileptic drug dyclonine, DMF, N-acetyl cysteine, and omaveloxolone among others, showed beneficial effects and frataxin-inducing activity in different models of FRDA (Petrillo et al., 2017; Clay et al., 2019; Petrillo et al., 2019). In addition, omaveloxolone was tested in a phase II clinical trial with 69 FRDA patients. In this study, the drug was overall well tolerated, but it did not change the primary outcome of the trial that is, peak workload in maximal exercise. However, it improved the modified Friedreich’s Ataxia Rating Scale (mFARS) that involves the examination of the neurological signs of the disease (Lynch et al., 2019a). In a follow-up double-blind, randomized, placebo-controlled, parallel-group, phase II trial performed with 103 FRDA patients from 11 institutions of the United States, Europe, and Australia (Trial number: NCT02255435, https://clinicaltrials.gov/ct2/show/NCT02255435), omaveloxolone significantly improved the neurological function of the FRDA patients compared to the placebo and was safe and well tolerated (Lynch et al., 2021), pointing to this drug as a potential therapeutic agent for FRDA.

Alpha-tocotrienol quinone (also known as Epi-743 or vatiquinone) is a molecule that blocks the activity of 15-lipoxygenase, an important oxidoreductase that regulates oxidative stress and neuroinflammation. Epi-743 received orphan drug designation for the treatment of mitochondrial epilepsy and other mitochondrial genetic diseases such as Leigh disease and Rett syndrome (Enns et al., 2012; Kahn-Kirby et al., 2019). Its safety and efficacy in improving visual and neurological functions in FRDA patients were also evaluated in a 6-month, double-blind, placebo-controlled, phase II clinical trial followed by 18 extra months of the open-label phase (Zesiewicz et al., 2018). In this study, the drug was shown to be safe and well tolerated by FRDA patients, but failed to improve key end points during the placebo phase. However, at the end of the 24-month intervention, EPI-743 significantly improved the neurological function and disease progression of the patients (Zesiewicz et al., 2018). In November 2020, PTC therapeutics launched MOVE-FA (Trial number: NCT04577352, https://clinicaltrials.gov/ct2/show/NCT04577352) a phase II/III double-blind, placebo-controlled trial with a follow-up open-label phase in children and adult FRDA patients from the United States, Canada, Europe, Australia, and Latin America to assess the efficacy and safety of vatiquinone. The study is currently ongoing. A total of 126 patients will be recruited.

Leriglitazone is the hydrochloride salt of the active metabolite M4 of pioglitazone. It is indicated to control glycemia in patients with type 2 diabetes. Leriglitazone, a PPAR-γ agonist with good brain penetration, is found to rescue neurodegeneration due to frataxin deficiency in the dorsal root ganglion neurons through restoration of mitochondrial membrane potential and improved mitochondrial function and calcium homeostasis (Rodriguez-Pascau et al., 2021). The drug also improved motor function in a mouse model of FRDA (Rodriguez-Pascau et al., 2021). In March 2019, leriglitazone received orphan designation from the EMA and the FDA for FRDA treatment. A phase II, randomized, double-blind, placebo-controlled clinical study called FRAMES (Trial number: NCT03917225, https://clinicaltrials.gov/ct2/show/NCT03917225?term=MIN102) has been launched to assess the efficacy and safety of leriglitazone in 36 FRDA patients. The trial is currently ongoing.

Interferon gamma is a drug currently approved in the United States for the treatment of chronic granulomatous disease and malignant osteoporosis (Britti et al., 2018). In the context of FRDA, this molecule increased frataxin expression in *in vitro* and *in vivo* models of the disease and enhanced motor function in mice (Wells et al., 2015). An initial pilot clinical trial with a small number of FRDA patients failed to detect an increase in frataxin expression but reported an improvement in the neurological outcome of the patients (Seyer et al., 2015). Nevertheless, a follow-up, double-blind, placebo-controlled study of interferon gamma performed in the US failed to replicate these findings since no difference in mFARS and frataxin levels was detected between the interferon gamma and the placebo groups (Lynch et al., 2019b).

Histone deacetylase inhibitors (HDACi) are anticancer agents that play important roles in epigenetic and non-epigenetic gene regulation. In the context of FRDA, preclinical *in vitro* and *in vivo* experiments provided proof of concept that these molecules can induce frataxin expression in different models of FRDA (Herman et al., 2006; Chan et al., 2013; Soragni et al., 2014). An initial exploratory, open-label, dose-escalation study with nicotinamide, a class III HDACi in FRDA patients, showed that the drug was safe and well tolerated. The study also showed a dose-dependent increase in frataxin mRNA expression in peripheral blood cells, together with a decrease in heterochromatin formation in the frataxin locus during the 8 weeks of daily nicotinamide dosing (Libri et al., 2014). In addition, a phase Ib clinical trial was performed with the HDACi 109/RG2833. In this study, the drug was safe and relatively well tolerated and induced a moderate increase in frataxin mRNA expression in peripheral blood cells (Soragni et al., 2014; Soragni and Gottesfeld, 2016). However, this HDAC inhibitor has poor brain penetration, and the active molecule is converted into inactive and potentially toxic metabolic products, pointing to the need of pharmacologic optimization to improve efficacy and reduce toxicity before embarking in prolonged clinical trials (Soragni and Gottesfeld, 2016).

The screening of a chemical library of 2000 FDA-approved compounds identified resveratrol, a naturally occurring antioxidant (Li et al., 2013), as a frataxin-inducing molecule with therapeutic potential for FRDA. In this study, HeLa cells expressing *FXN-EGFP* were used to identify molecules that are able to induce frataxin expression (Li et al., 2013). Among the 18 compounds identified as positive hits, resveratrol was the one with the best frataxin-inducing capacity in fibroblasts and lymphoblasts from FRDA patients, and the one having lesser toxic cell effects (Li et al., 2013). An open-label, non-randomized, phase I clinical trial with resveratrol failed to increase frataxin levels in FRDA patients; however, high doses of resveratrol were associated with positive clinical outcomes (Yiu et al., 2015). In May 2019, a double-blind, placebo-controlled, 2-period crossover phase 2 trial with micronized resveratrol (Trial number: NCT03933163, https://clinicaltrials.gov/ct2/show/NCT03933163) was launched to measure the neurological impact of this drug. The trial is still ongoing.

Table 2 provides additional information about the aforementioned drugs, including pharmaceutical companies or academic institutions involved in designing and running the clinical trials, and whether the drugs received orphan designation from the EMA and/or the FDA, and the sponsors that made the orphan designation request.

**TABLE 2:** List of the repurposed drugs, with and without orphan designation or drug marketing authorization for Friedreich’s ataxia (FRDA) mentioned in this article. The sponsors (entities involved in making the orphan designation request to EMA or the FDA), and the public or private organizations involved in designing and running the clinical trials are detailed. N/A = non-applicable.

Drug name	Orphan drug designation for FRDA?	Sponsor	Information about the sponsors	Drug marketing authorization for FRDA?	Original indication of the drug	Mechanism of action and preclinical findings	Outcome of the clinical trials	Entities involved In designing and running the clinical trials	References
Deferiprone (Ferriprox^®^)	Yes: FDA, 2008	*US:* Chiesi United States, Inc	Pharmaceutical company generating products for hospitals, adjacent specialties, and rare disease markets	No	Thalassemia syndrome	Iron chelator. The drug reduced mitochondrial iron overload in FRDA models	The drug was safe and well tolerated at lower doses in a phase II, double-blind placebo-controlled study with FRDA patients. A moderate improvement in cardiac hypertrophy but no neuroprotection was observed	Trials sponsored by ApoPharma Inc. (which was acquired by Chiesi Farmaceutici S.p.A in 2020). In collaboration with academic institutions and hospitals from Belgium, France, Italy, Spain, Canada, and Australia	Pandolfo and Hausmann. (2013); Pandolfo et al. (2014) https://clinicaltrials.gov/ct2/show/NCT00897221?cond=Friedreich+Ataxia&draw=2&rank=12 https://clinicaltrials.gov/ct2/show/NCT00530127?cond=Friedreich+Ataxia&draw=2&rank=21
Idebenone (Raxone^®^)	Yes: EMA, 2001 and 2004; FDA, 2004	*Europe:* 2001 orphan designation given to Laboratories Takeda, France *. 2004 orphan designation given to Promedipharm GmbH, Germany. In 2005 this orphan designation was transferred to Santhera Pharmaceuticals, Deutschland &. *US:* Santhera Pharmaceuticals, Switzerland §	* Big Pharmaceutical company. & Multinational clinical trial company. § Multinational clinical trial company	No	Leber’s hereditary optic neuropathy	Antioxidant. The drug reduced cell death in frataxin-deficient cells and delayed the onset of cardiac dysfunction in a mouse model of the disease	An initial clinical trial with this drug suggested some cardioprotective effects. No significant neuro or cardioprotective effects were seen on several follow-up clinical studies. Only a single open-label study showed some neurological improvement	Trials sponsored by Santhera Pharmaceuticals, or the NIH National Institute of Neurological Disorders and Stroke (NINDS), or Assistance Publique - Hôpitaux de Paris, in collaboration with various academic institutions and hospitals from the US, Europe, and the United Kingdom	Rustin et al. (1999); Hausse et al. (2002); Jauslin et al. (2002); Buyse et al. (2003); Mariotti et al. (2003); Rustin et al. (2004); Seznec et al. (2004); Rinaldi et al. (2009); Lynch et al. (2010); Meier et al. (2012)
									https://clinicaltrials.gov/ct2/show/NCT00697073?cond=Friedreich+Ataxia&draw=2&rank=34
									https://clinicaltrials.gov/ct2/show/NCT00537680?cond=Friedreich+Ataxia&draw=2&rank=38
									https://clinicaltrials.gov/ct2/show/NCT01303406?cond=Friedreich+Ataxia&draw=2&rank=41
									https://clinicaltrials.gov/ct2/show/NCT00229632?cond=Friedreich+Ataxia&draw=2&rank=47
									https://clinicaltrials.gov/ct2/show/NCT00905268?cond=Friedreich+Ataxia&draw=2&rank=51
									https://clinicaltrials.gov/ct2/show/NCT00078481?cond=Friedreich+Ataxia&draw=2&rank=55
									https://clinicaltrials.gov/ct2/show/NCT00015808?cond=Friedreich+Ataxia&draw=2&rank=58
									https://clinicaltrials.gov/ct2/show/NCT00993967?cond=Friedreich+Ataxia&draw=2&rank=64
									https://clinicaltrials.gov/ct2/show/NCT00224640?term=idebenone&cond=Friedreich+Ataxia&draw=2&rank=9
Omaveloxolone (RTA-408)	Yes: EMA, 2018; FDA, 2017	*Europe:* In 2018, orphan designation was given to Dr Stefan Blesse (Principal Consultant), in 2019 designation was transferred to Granzer Regulatory Consulting & Services, Germany *, and in 2019 to Reata Ireland Limited §. *US:* Reata Pharmaceuticals Inc., US $	*Consulting. § $ Pharmaceutical companies	No	Anticancer drug indicated for non–small cell lung cancer	Compound with antioxidant and anti-inflammatory properties. In FRDA models, this drug prevents NRF2 ubiquitination and degradation improving mitochondrial function and reducing oxidative stress and enhancing frataxin expression	The drug was safe and well tolerated in a double-blind, randomized, placebo-controlled, phase II study. A significant improvement in patient neurological functions was observed (MOXIe)	Trial sponsored by Reata Pharmaceuticals, Inc. with the collaboration of AbbVie (Private Biopharmaceutical laboratory), FARA (patient association), and 11 academic institutions and hospitals from the US, Australia, Italy, Austria, and the United Kingdom.	Petrillo et al. (2017); Lynch et al. (2019a); Clay et al. (2019); Petrillo et al. (2019); Lynch et al. (2021)
									https://clinicaltrials.gov/ct2/show/NCT02255435
Vatiquinone (Epi-743)	Yes: EMA, 2021; FDA, 2014	*Europe:* PTC Therapeutics International Limited, Ireland *. *US:* PTC Therapeutics, Inc., US §	Global diversified biopharmaceutical company developing drug and small-molecule therapies for rare genetic disorders and serious diseases. *International Headquarters of the company. § Corporate Headquarters	No	The drug received orphan designation for mitochondrial epilepsy, Leigh disease, and RARS2 syndrome but did not receive marketing authorization yet	Compound with lipooxygenase blocker activity. It regulates oxidative stress and neuroinflammation in FRDA	An initial clinical trial showed that the drug was safe and well tolerated. An improvement in patient’s neurological function and disease progression was observed. A follow-up phase II/III, double-blind, placebo-controlled trial is currently ongoing (MOVE-FA)	Trials sponsored by the University of South Florida, or PTC therapeutics with the collaboration of Edison Pharmaceuticals, FARA (patients’ association) and and 14 academic institutions and hospitals from the US, France, Germany, Italy, Spain, Brazil, Canada, Australia, and New Zealand	Zesiewicz et al. (2018) https://clinicaltrials.gov/ct2/show/NCT01962363?cond=Friedreich+Ataxia&draw=2&rank=5
									https://clinicaltrials.gov/ct2/results?cond=Friedreich+Ataxia&term=&cntry=&state=&city=&dist=
									https://clinicaltrials.gov/ct2/show/NCT01370447?cond=Friedreich+Ataxia&draw=2&rank=73
									https://clinicaltrials.gov/ct2/show/NCT04577352?cond=Friedreich+Ataxia&draw=2&rank=22
Leriglitazone (MIN-102) Minoryx^®^	Yes, EMA, 2019; FDA, 2019	*Europe and US*: Minoryx Therapeutics S.L, Spain	Clinical stage biotech company with a focus on orphan diseases	No	Leriglitazone is the hydrochloride salt of the active metabolite M4 of pioglitazone, indicated to control glycemia in patients with type 2 diabetes	Compound with PPAR-γ agonist activity. The drug rescues neurodegeneration in FRDA through restoration of mitochondrial membrane potential, improvement of mitochondrial function, and calcium homeostasis. It also improved motor function in frataxin -deficient mice	A phase II, randomized, double-blind, placebo-controlled clinical study is currently ongoing (FRAMES)	Minoryx Therapeutics (sponsor) together with academic hospitals from Belgium, France, Germany, and Spain	Rodriguez-Pascau et al. (2021) https://www.clinicaltrials.gov/ct2/show/NCT03917225
Interferon gamma (Actimmune^®^) (Imukine^®^)	Yes, EMA, 2011; FDA 2011 and 2014	Europe: orphan designation first given to Prof. Roberto Testi, Dept. of Experimental Medicine, Univ. of Rome “Tor Vergata, Italy *; designation transferred to Horizon Pharma Ireland Limited, Ireland & US: Prof. Roberto Testi, Dept. of Experimental Medicine, Univ. of Rome “Tor Vergara, Italy *	*Academia. & Global biotechnology company	No	Chronic granulomatous disease and malignant osteoporosis	Treatment with this drug results in STAT1 phosphorylation, nuclear translocation, and initiation of gene transcription of multiple immune-related genes. Upregulates frataxin protein expression in *in vitro* and *in vivo* models of FRDA and enhanced motor function in frataxin-deficient mice	A phase II, randomized, double-blind, placebo-controlled study failed to show differences in mFARS and frataxin levels among controlled and placebo groups	Trials sponsored by Horizon Pharma Ireland, Ltd., or Children’s Hospital of Philadelphia, US or Azienda Policlinico Umberto I, Italy, or IRCCS Eugenio Medea, Italy (Research Institute) in collaboration with FARA (patient association), Vidara Therapeutics Research Ltd., and several academic institutions and hospitals from the US	Seyer et al. (2015); Wells et al. (2015); Lynch et al. (2019b) https://clinicaltrials.gov/ct2/show/NCT01965327?cond=Friedreich+Ataxia&draw=2&rank=7
									https://clinicaltrials.gov/ct2/show/NCT02035020?cond=Friedreich+Ataxia&draw=2&rank=28
									https://clinicaltrials.gov/ct2/show/NCT02797080?cond=Friedreich+Ataxia&draw=2&rank=33
									https://clinicaltrials.gov/ct2/show/NCT02593773?cond=Friedreich+Ataxia&draw=2&rank=36
									https://clinicaltrials.gov/ct2/show/NCT02415127?cond=Friedreich+Ataxia&draw=2&rank=39
									https://clinicaltrials.gov/ct2/show/NCT03888664?term = interferon+gamma&cond = Friedreich+Ataxia&draw = 2&rank = 6
Resveratrol	Yes: FDA, 2017	Jupiter Orphan Therapeutics, US	Is a clinical-stage CNS and rare disease-focused company	No	Indicated in cancer, heart diseases, inflammation and immunity, diabetes, and viral infection	Naturally occurring antioxidant. Activates sirtuin 1 by modulating the activity of PPAR-γ and PGC-1α. In FRDA models, it upregulated frataxin expression	No clinical data are available yet. A phase II, randomized, blinded, placebo-controlled crossover trial with micronized resveratrol to assess the neurological effects of the drug in FRDA patients was started in 2019 and is still ongoing	The trial is sponsored by the Murdoch Childrens Research Institute, Australia with the collaboration of FARA (patient’s association) and several academic institutions and hospitals from Australia	Li et al. (2013); Yiu et al., 2015)
									https://clinicaltrials.gov/ct2/show/NCT01339884?term=resveratrol&cond=Friedreich+Ataxia&draw=2&rank=2
									https://clinicaltrials.gov/ct2/show/NCT03933163?term=resveratrol&cond=Friedreich+Ataxia&draw=2&rank=1
HDAC inhibitors N-(6-(2-aminophenylamino)-6-oxohexyl)-4-methylbenzamide (RG2833, RGFP109)	Yes: EMA, 2010; FDA, 2010	*Europe: Repligen Europe Limited, Ireland*. *US: Repligen Corporation*. https://www.prnewswire.com/news-releases/repligen-receives-orphan-drug-designation-from-the-fda-for-rg2833-for-friedreichs-ataxia-94737209.html	Bioprocessing-focused life sciences company	No	Anti-cancer drug	Histone deacetylase inhibitor. In preclinical studies, these drugs induced frataxin mRNA expression by epigenetic gene regulation	An exploratory, open-label, dose-escalation study with nicotinamide, a class III HDACi, showed that the drug is safe and well tolerated and can induce frataxin mRNA expression. In a phase Ib study, another HDACi, 109/RG2833, was safe and well tolerated and also resulted in a moderate increase in frataxin mRNA levels, and H3 lysine 9 acetylation in peripheral blood mononuclear cells was observed. This HDACi, however, has low brain penetration. Pharmacologic optimization is needed before performing other trials	Trial sponsored by RWTH Aachen University; or the Imperial College of London; or Repligen corporation, in collaboration with Assistance Publique - Hôpitaux de Paris, Fara (patient association), and academic institutions and hospitals from Austria, France, Germany, Italy, Spain, and the United Kingdom	Herman et al. (2006); Chan et al. (2013); Libri et al. (2014); Soragni et al. (2014); Soragni and Gottesfeld. (2016)
									https://clinicaltrials.gov/ct2/show/NCT03761511?term=histone&cond=Friedreich+Ataxia&draw=2&rank=2
									https://clinicaltrials.gov/ct2/show/NCT01589809?term=nicotinamide&cond=Friedreich+Ataxia&draw=2&rank=2
Etravirine Intelence^®^	No	N/A	N/A	No	Anti-HIV drug	In preclinical studies, this drug promoted frataxin mRNA translation restoring physiological frataxin levels. This results in the improved Fe–S cluster biogenesis, mitochondrial function, and reduced oxidative stress in frataxin-deficient cells	A phase II, open-label pilot, study is currently ongoing to evaluate its safety and efficacy in FRDA patients	Trial sponsored by the IRCCS Medea Scientific Institute, Italy in collaboration with the University of Rome Tor Vergata, Italy	Alfedi et al. (2019) https://clinicaltrials.gov/ct2/show/NCT04273165?cond=Friedreich+Ataxia&draw=2&rank=18
GLP-1 analogs Exenatide (Byetta^®^)	No	N/A	N/A	No	Type 2 diabetes	cAMP inducer. In preclinical studies, the drug improved pancreatic β-cell function, reduced apoptosis and oxidative stress and increased mitochondrial function in frataxin-deficient β-cells and neurons. It also induced frataxin protein expression in both cell types *in vitro* and *in vivo*	A pilot study with GLP-1 analogs in FRDA patients showed that the drug does not induce major adverse events. A modest platelet frataxin induction upon exenatide administration was observed in this pilot trial	The pilot trial was sponsored by the Université Libre de Bruxelles, Belgium in and FARA (patient association)	Cnop et al. (2012); Igoillo-Esteve et al. (2015); Igoillo-Esteve et al. (2019)
									https://www.clinicaltrialsregister.eu/ctr-search/trial/2014-003598-41/BE

The following websites were consulted to build up this table. Community Register of orphan medicinal products from the European commission: https://ec.europa.eu/health/documents/community-register/html/reg_od_act.htm?sort=a; Orphan Drug Designations and Approvals from the FDA: https://www.accessdata.fda.gov/scripts/opdlisting/oopd/.ClinicalTrial.gov to retrieve the list of clinical trials for FRDA: https://clinicaltrials.gov/ct2/results?cond=Friedreich+Ataxia&term=&cntry=&state=&city=&dist=.

#### Additional Drug Repurposing–Based Therapeutic Strategies Under Investigation

Etravirine, an antiviral drug currently in use as an anti-human immunodeficiency virus therapy, was identified as a potential frataxin-inducing molecule during the screening of a library of 853 US FDA–approved compounds using a high-throughput cell-based reporter assay to monitor variations in frataxin levels (Alfedi et al., 2019). Of the 853 compounds examined, 19 were able to promote at least 2-fold increase in frataxin levels. From those, etravirine was the most potent frataxin inducer in cells derived from FRDA patients (Alfedi et al., 2019). Indeed, this molecule was able to importantly induce frataxin precursor levels by selectively enhancing the translation efficiency of frataxin transcripts by promoting a shift of frataxin mRNA from silent isolated ribosomes toward translationally active polysomal subsets. This resulted in an increase in the frataxin levels to the ones present in unaffected carriers and restoration of aconitase activity (Alfedi et al., 2019). Based on these promising results, in September 2020, a phase II clinical study had been launched (Trial Number: NCT04273165, https://clinicaltrials.gov/ct2/show/NCT04273165) to evaluate the safety and efficacy of etravirine in FRDA patients.

Glucagon-like peptide 1 (GLP-1) analogs are drugs currently used for the treatment of type 2 diabetes (Vilsboll and Knop, 2008). They stimulate cAMP formation by binding to G protein-coupled receptors resulting in the activation of intracellular signaling pathways. In pancreatic β-cells, these drugs improve insulin synthesis and secretion and prevent apoptosis (Drucker et al., 1987; Yusta et al., 2006; Cunha et al., 2009). Besides being present in β-cells, GLP-1 receptors are also expressed in the heart and brain (Campbell and Drucker, 2013), and it was shown that GLP-1 analogs have cardiovascular (Ban et al., 2008) and neuroprotective actions (McClean et al., 2011). In the context of FRDA, the cAMP inducer forskolin and the GLP-1 analog exenatide were shown to reduce apoptosis in frataxin-deficient β-cells and neurons by decreasing oxidative stress and inhibiting the mitochondrial pathway of apoptosis (Cnop et al., 2012; Igoillo-Esteve et al., 2015). Besides having this protective effect, it was recently demonstrated that GLP-1 analogs and cAMP inducers also improve the functionality of pancreatic β-cells and reduce mitochondrial dysfunction in patient-derived sensory neurons (Igoillo-Esteve et al., 2019). In addition, it was demonstrated that GLP-1 analogs and cAMP inducers enhance frataxin protein expression in *in vitro* and *in vivo* FRDA models and in a pilot study with FRDA patients (Igoillo-Esteve et al., 2019). Altogether these data provide a strong rationale for the design of a long-term clinical trial to assess the disease-modifying effect of GLP-1 analogs in FRDA patients. The characteristics of these drugs have been summarized in Table 2.

### Friedreich Ataxia Patient Associations and Foundations

The Friedreich’s Ataxia Research Alliance (FARA, https://www.curefa.org) is a non-profit, voluntary organization that partners with government agencies, corporations, and advocacy groups to support scientific research focused on the development of therapeutic strategies to stop the advancement of or cure FRDA.

### Wolfram Syndrome

#### Clinical Features and Genetic Cause

**FIGURE 3:**
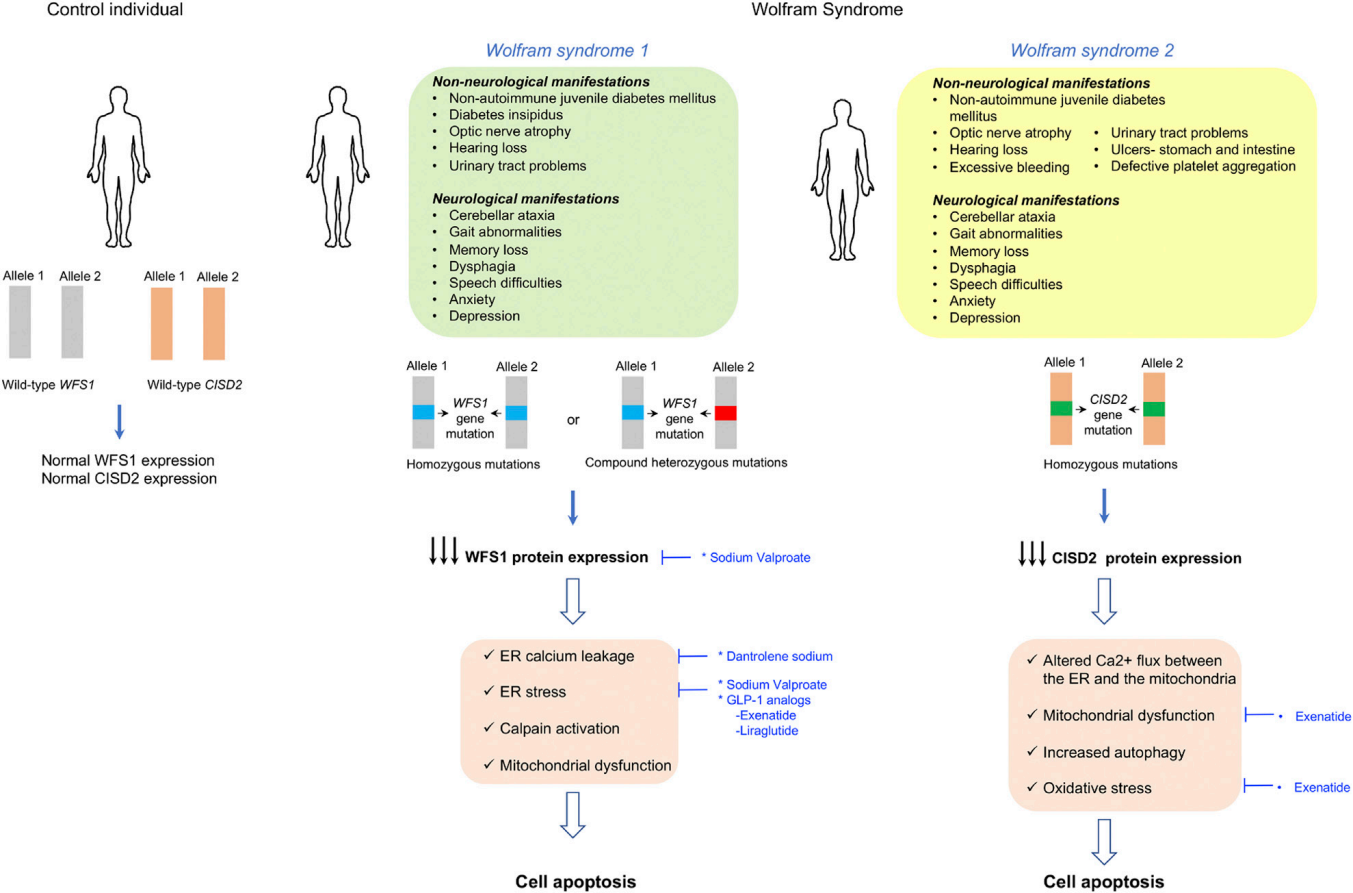
Clinical manifestations and molecular disease mechanisms in Wolfram syndrome. Wolfram syndrome 1 is caused by homozygous or heterozygous mutations in the *WFS1* gene, while wolfram syndrome 2 is caused by mutations in the *CISD2* gene. This results in reduced WFS1 or CISD2 protein expression, which causes several cell defects leading to cell dysfunction and apoptosis which result in the clinical manifestations of the disease. Repurposed drugs with and without orphan designation for Wolfram syndrome are highlighted in blue along with the cellular dysfunctions or clinical manifestations that they tackle. ER- endoplasmic reticulum, GLP-1- glucagon-like peptide-1.

Wolfram syndrome is a rare autosomal life-threatening disease with a frequency of 1/160,000 to 1/770,000 individuals in the United States and United Kingdom, respectively (Fraser and Gunn, 1977; Barrett et al., 1995). Two types of Wolfram syndrome exist that share a large number of clinical manifestations: Wolfram syndrome 1 and Wolfram syndrome 2 (Rigoli and Di Bella, 2012). The former is the most common. The majority of Wolfram syndrome 1 patients have biallelic homozygous or compound heterozygous mutations in the *WFS1* gene (Inoue et al., 1998; Hardy et al., 1999; Gomez-Zaera et al., 2001; Khanim et al., 2001; Cryns et al., 2003; Hansen et al., 2005); however, autosomal dominant inherited or *de novo* mutations in the same gene have also been reported in some individuals (Bespalova et al., 2001; Eiberg et al., 2006; Hogewind et al., 2010; Bonnycastle et al., 2013; De Franco et al., 2017). Wolfram syndrome 2 is caused by mutations in the *CISD2* gene (Rigoli and Di Bella, 2012; Mozzillo et al., 2014). Wolfram syndrome 1, also known as DIDMOAD, is characterized by non-autoimmune juvenile diabetes mellitus, diabetes insipidus, optic nerve atrophy, hearing loss, urinary tract problems, and progressive neurodegeneration that manifests principally as cerebellar ataxia, gait abnormalities, memory loss, dysphagia, speech difficulties, anxiety, and depression (Rando et al., 1992; Barrett et al., 1995; Kinsley et al., 1995; Marshall et al., 2013). Wolfram syndrome 2 patients have similar clinical manifestations with the exception that they do not develop diabetes insipidus, and they have stomach and intestine ulcers, defective platelet aggregation, and excessive bleeding (El-Shanti et al., 2000; Rigoli and Di Bella, 2012; Mozzillo et al., 2014; Rondinelli et al., 2015). Both forms of Wolfram syndrome have poor prognosis, and the patients die prematurely at a median age of 30 years due to progressive severe neurological dysfunction and respiratory failure resulting from brain stem atrophy (Urano, 2016). Both forms of the disease are progressive and exhibit a clear chronology of clinical manifestations (Figure 3).

### Molecular Phenotype

The *WFS1* gene encodes an endoplasmic reticulum (ER) transmembrane protein called wolframin or WFS1 that is highly expressed in the brain, pancreas, heart, lungs, liver, and kidneys (Yamada et al., 2006). The ER is an essential organelle for secretory cells, such as pancreatic β-cells and neurons, since most secretory proteins are synthesized, folded, and modified within the ER before being transported into the Golgi for secretion (Schwarz and Blower, 2016). It has been demonstrated that WFS1 deficiency causes ER stress in pancreatic β-cells, neurons, retinal ganglion cells, and oligodendrocytes, resulting in dysfunction and degeneration of the affected tissues (Ishihara et al., 2004; Fonseca et al., 2005; Riggs et al., 2005; Fonseca et al., 2010; Fonseca et al., 2011; Fischer and Ehrlich, 2020). WFS1 regulates ER calcium homeostasis and ER stress by interacting with the sarcoplasmic ER calcium (SERCA) pump and the ER stress transducer ATF6, respectively (Fonseca et al., 2005; Zatyka et al., 2008; Fonseca et al., 2009; Fonseca et al., 2010; Fonseca et al., 2011; Hatanaka et al., 2011; Lu et al., 2014; Zatyka et al., 2015). Accordingly, WFS1-deficiency results in ER calcium depletion, enhanced cytosolic Ca^2+^, calpain activation, and cell death (Lu et al., 2014). Moreover, it was also demonstrated that WFS1 regulates secretory granule acidification in β-cells and neurons (Gharanei et al., 2013; Sutt et al., 2015) and that the ER dysfunction caused by WFS1 deficiency is accompanied by altered mitochondrial function in β-cells and neurons contributing to diabetes and neurodegeneration (Cagalinec et al., 2016; Maxwell et al., 2020). Using a rat model of the disease, it was recently demonstrated that the lack of functional WFS1 alters calcium homeostasis in cardiac myocytes as a result of reduced expression of the plasmalemmal sodium–calcium exchanger type 1 (NCX1) (Kurekova et al., 2020).

Regarding Wolfram syndrome 2, the *CISD2* gene encodes a highly conserved zinc finger Fe–S cluster containing a protein called CISD2 or Miner1. This protein is localized in the ER and in the mitochondrial-associated membranes (MAMs) (Amr et al., 2007). Its function is still unknown, but it has been proposed that it plays an important role in iron donation to the mitochondria, regulation of oxidative stress, and preservation of mitochondrial and ER Ca^2+^ homeostasis (Wiley et al., 2013; Wang et al., 2014). *CISD2* deficiency alters the Ca^2+^ flux between the ER and the mitochondria resulting in mitochondrial dysfunction and reduced mitochondrial integrity, that is accompanied by an upregulation of autophagy and pro-apoptotic factors (Chen et al., 2009; Sohn et al., 2013; Danielpur et al., 2016; Holt et al., 2016). Despite the exact mechanism not being known, preclinical experiments in several Wolfram syndrome 2 cases showed that *CISD2* deficiency leads to neuronal and β-cell death, a process probably mediated by calpain activation (Lu et al., 2014) (Figure 3).

There are currently no approved therapeutic options to prevent, delay, or cure Wolfram syndrome, but numerous drug repurposing–based approaches are currently under investigation to manage the clinical manifestations of the disease. Since ER stress is deleterious for pancreatic β-cells and neurons, and is a hallmark of Wolfram syndrome, it has been proposed that reducing ER stress may have beneficial outcomes in this life-threatening disease. Accordingly, different ER stress–targeting approaches are being tested, for example, ER calcium stabilizers, chemical chaperones, GLP-1 analogs, and modulators of ER stress (Urano, 2016; Abreu et al., 2021; Pallotta et al., 2019).

### Approved and Non-Approved Orphan-Designated Repurposed Drugs for Wolfram Syndrome

As mentioned above, there are currently no approved drugs for the treatment of Wolfram syndrome; however, two repurposed medical compounds, namely, dantrolene sodium and sodium valproate received orphan designation for this disease (https://www.orpha.net).

Dantrolene sodium was initially approved by the FDA for malignant hyperthermia and muscle spasms derived from spinal cord injury, stroke, cerebral palsy, or multiple sclerosis. This drug acts as an ER calcium stabilizer by inhibiting ryanodine receptors on the ER (Fruen et al., 1997). In the context of Wolfram syndrome, preclinical studies using different models of the disease showed that dantrolene is able to suppress β-cell and neuronal death by preventing calcium leakage from the ER (Lu et al., 2014). This drug received orphan designation for Wolfram syndrome from EMA and the FDA. An open-label, phase Ib/IIa trial in pediatric and adult Wolfram syndrome patients with dantrolene sodium was recently performed (Abreu et al., 2021). The primary objective of the study was to assess the safety and tolerability of the molecule, and the secondary objectives were to evaluate the efficacy of the treatment in improving residual pancreatic β-cell function, visual acuity, quality of life, and measures related to vision and neurological functions. The study showed that dantrolene sodium is well tolerated by Wolfram syndrome patients, but β-cell function, visual acuity, and neurological functions were not significantly improved after 6 months of treatment (Abreu et al., 2021). However, a patient subgroup analysis revealed a significant improvement in β-cell function in subjects who possessed the greatest degree of β-cell function at the baseline. Moreover, the inflammation markers IL-1β and IL-21, that are increased in Wolfram syndrome patients as a result of ER stress (Oslowski et al., 2012), were significantly decreased in dantrolene-treated subjects (Abreu et al., 2021). These results suggest that this molecule may be beneficial in treating certain manifestations of the disease and justifies further investigation in using dantrolene sodium and other small molecules targeting the ER for the treatment of Wolfram syndrome.

Sodium valproate is a drug indicated to treat different neuropsychiatric disorders, such as epilepsy, bipolar disorder, and migraine. This molecule exerts its beneficial effects through multiple mechanisms of action (Johannessen and Johannessen, 2003; Rosenberg, 2007). In the context of Wolfram syndrome, sodium valproate was shown to increase WFS1 mRNA expression in neuronal cells by activating its promoter. Moreover, this drug was shown to enhance the dissociation of WFS1 from GRP94, suggesting that it may have ER stress–modulating effects (Kakiuchi et al., 2009). A recent study showed that sodium valproate also reduces ER stress and cell apoptosis in Wolfram syndrome 1 models caused by dominant *WFS1* mutations (Batjargal et al., 2020). This drug received orphan designation for Wolfram syndrome from the EMA and the FDA. A phase II, placebo-controlled clinical trial with 70 pediatric and adult Wolfram syndrome patients has been launched (Trial Number: NCT03717909, https://clinicaltrials.gov/ct2/show/study/NCT03717909). The aim of this study is to assess the efficacy, safety, and tolerability of sodium valproate in the treatment of Wolfram syndrome patients. The primary outcome of this trial is visual acuity, and the secondary outcomes are safety, tolerability, and neurological outcomes.

Table 3 provides additional information about the aforementioned drugs, including pharmaceutical companies or academic institutions involved in designing and running the clinical trials, and whether the drugs received orphan designation from the EMA and/or the FDA, and the sponsors that made the orphan designation request.

**TABLE 3:** List of the repurposed drugs, with and without orphan designation or drug marketing authorization for Wolfram syndrome (WS) mentioned in this article. The sponsors (entities involved in making the orphan designation request to the EMA or the FDA), and the public or private organizations involved in designing and running the clinical trials are detailed. N/A = non-applicable.

Drug name	Orphan drug designation for WS?	Sponsor	Information about the sponsors	Drug marketing authorization for WS?	Original indication of the drug	Mechanism of action and preclinical findings	Outcome of the clinical trials	Entities involved in designing and running the clinical trials	References
Sodium valproate Depakine^®^	Yes: EMA, 2015; FDA, 2015	*Europe:* Orphan designation given to Alan Boyd Consultants Ltd., Electra House, United Kingdom in 2015. In 2019, the designation was transferred to Boyd Consultants Limited, Dublin, Ireland *. *US:* University of Birmingham, United Kingdom §	*Consulting. § University	No	Epilepsy, bipolar disorder, and migraine	The exact mechanism of action is unknown. In preclinical models of wolfram syndrome, it increased WFS1 mRNA expression in neuronal cells and acted as an ER stress modulator	A phase II, double blind, placebo-controlled, clinical trial is ongoing to evaluate the safety, efficacy, and tolerability of the drug in pediatric and adult patients with Wolfram syndrome. (Clinical Trial Number: NCT03717909)	The trial is sponsored by the University of Birmingham, United Kingdom, in collaboration with Wolfram syndrome United Kingdom (patient’s association) and academic institutions and hospitals from France, Poland, Spain, and the United Kingdom.	Kakiuchi et al. (2009)
									https://clinicaltrials.gov/ct2/show/NCT03717909?cond=Wolfram+syndrome&draw=2&rank=1
									https://clinicaltrials.gov/ct2/show/NCT04940572?cond=Wolfram+syndrome&draw=2&rank=2
Dantrolene sodium Dantrium^®^	Yes: EMA, 2016; FDA 2016	*Europe:* Orphan designation given to Alan Boyd Consultants Ltd., Electra House, United Kingdom in 2015. In 2019, the designation was transferred to Boyd Consultants Limited, Dublin, Ireland *. *US:* Washington University in St. Louis, US §	*Consulting § Academia	No	Malignant hyperthermia and muscle spasm derived from spinal cord injury, stroke, cerebral palsy, or multiple sclerosis	ER calcium stabilizer. In Wolfram syndrome preclinical models, the drug suppressed β-cell and neuronal death by preventing calcium leakage from the ER.	The drug was found to be safe and well-tolerated in an open-label, phase Ib/IIa study; however, β-cell function, visual activity, and neurological functions were not significantly improved. In a stratified analysis a sub-group of patients exhibited improvement in β-cell function	Trial sponsored by the Washington University School of Medicine, US in collaboration with the NIH. National Institute of Diabetes and Digestive and Kidney Diseases (NIDDK), the Snow Foundation (patient association), and Ellie White Foundation (patient association)	Lu et al. (2014); Abreu et al. (2021)
									https://clinicaltrials.gov/ct2/show/NCT02829268?cond=Wolfram+syndrome&draw=2&rank=5
GLP-1 analogs Liraglutide Victoza^®^ Exenatide Byetta^®^	No	N/A	N/A	No	Type 2 diabetes	cAMP inducer through the activation of the G-protein coupled receptor. Preclinical studies in animal models of Wolfram syndrome showed that these drugs improved β-cell function and reduced neuroinflammation and improved ER stress in Wolfram syndrome 1 and 2	GLP-1 agonist treatment significantly improved the glycemic control in one Wolfram syndrome 1 patient, and in a second patient with a dominant form of Wolfram syndrome. A clinical trial with exenatide in Wolfram syndrome 2 patients was launched, and a second one with Liraglutide in Wolfram syndrome 1 patients has been recently announced	The trial in Wolfram syndrome 2 was sponsored by Hadassah Medical Organization, Israel. The trial in Wolfram syndrome 1 is sponsored by the Snow Foundation (patient association) and the Washington University in St Louis, US	Kondo et al. (2018); Toots et al. (2018) Sedman et al. (2016); Seppa et al. (2019)
									https://clinicaltrials.gov/ct2/show/NCT01302327?cond=Wolfram+syndrome&draw=2&rank=4
									https://thesnowfoundation.org/trial-of-liraglutide-in-wolfram-syndrome/

The following websites were consulted to build up this table. Community Register of orphan medicinal products from the European commission: https://ec.europa.eu/health/documents/community-register/html/reg_od_act.htm?sort=a; Orphan Drug Designations and Approvals from the FDA: https://www.accessdata.fda.gov/scripts/opdlisting/oopd/. ClinicalTrial.gov to retrieve the list of clinical trials for WS: https://clinicaltrials.gov/ct2/results?cond=Wolfram+syndrome.

### Additional Drug Repurposing–Based Therapeutic Strategies Under Investigation

GLP-1 analogs also appear as a promising therapeutic opportunity for Wolfram syndrome (Table 3). As mentioned before, these molecules are used for the treatment of type 2 diabetes and were shown to alleviate ER stress (Cunha et al., 2009; Drucker, 2018) and enhance the expression of anti-apoptotic proteins (Yusta et al., 2006; Cunha et al., 2009). Moreover, some of them cross the blood–brain barrier and confer neuroprotection (Holst et al., 2011; Hunter and Holscher, 2012; Porter et al., 2013; Zhang et al., 2018). In addition, it was shown that topical administration of GLP-1 analogs prevents retinal neurodegeneration, suggesting that these molecules may be useful to treat diabetes, neurodegeneration, and blindness in Wolfram syndrome (Hernandez et al., 2016; Hernandez et al., 2017). Accordingly, it was shown that acute exenatide injection in a mouse model of the disease enhances insulin secretion (Sedman et al., 2016) and that prolonged exenatide and liraglutide administration in WFS1-deficient mice and rats prevent glucose intolerance and improve glucose-stimulated insulin secretion by reducing cellular stress (Kondo et al., 2018; Toots et al., 2018). Additionally, a follow-up study performed with WFS1-deficient rats showed that the 6-month liraglutide treatment in these animals reduced neuroinflammation and improved ER stress in the inferior olive (Seppa et al., 2019). Moreover, this drug protected retinal ganglion cells from cell death and optic nerve axons from degeneration, suggesting that GLP-1 analogs may, indeed, be beneficial in preventing neurodegeneration and vision loss (Seppa et al., 2019). GLP-1 agonist treatment significantly improved the glycemic control in a patient with a dominant form of Wolfram syndrome, suggesting that treatment with these drugs should also be considered in patients with dominant forms of Wolfram syndrome (Scully and Wolfsdorf, 2020).

Exenatide was shown to also be beneficial in Wolfram syndrome 2. Indeed, in a β-cell model of the disease, these drugs improved glucose-stimulated insulin secretion, reduced the accumulation of labile iron in the mitochondria, and alleviated oxidative stress. Moreover, exenatide administration in one Wolfram syndrome 2 patient resulted in a 70% reduction in daily insulin requirements, improved glycemic control, and 7-fold increase in maximal insulin secretion (Danielpur et al., 2016). Altogether, these results provide evidence of the important therapeutic potential of these drugs in Wolfram syndrome. Accordingly, a clinical trial with exenatide in Wolfram syndrome 2 (Trial Number: NCT010302327) was launched, and a second one with liraglutide (Victoza^®^) in Wolfram syndrome 1 patients has recently been announced by Washington University with the help of the Snow Foundation (https://thesnowfoundation.org/trial-of-liraglutide-in-wolfram-syndrome/). If these drugs are shown to slowdown or revert some of the clinical manifestations of the pathology, it will constitute a great advancement in the management of this life-threatening orphan disease.

### Wolfram Syndrome Patient Associations and Foundations

Many Wolfram syndrome patient associations exist that importantly contribute to fund preclinical and clinical research projects focused on the development of therapeutic opportunities for the disease. The Wolfram Syndrome Research Alliance (WSRA) (https://www.wsresearchalliance.org/foundations-supporting-ws-research.html) serves as a centralized portal to connect and coordinate the efforts of researchers, clinicians, and governmental and non-profit agencies to accelerate the development of effective treatments. This portal also provides the full list of existing Wolfram syndrome patient associations, the research groups currently working on the disease, and a pipeline of the potential therapies under study. Accordingly, different studies based on Wolfram Syndrome are funded by various patient advocacy groups. As mentioned in some examples, the clinical trial of sodium valproate (NCT03717909) initiated in October 2018, is supported by Wolfram Syndrome United Kingdom https://www.findacure.org.uk/drug-repurposing and United Kingdom Research and Innovation (UKRI) https://gtr.ukri.org/projects?ref=MR%2FP007732%2F1, the trial with dantrolene sodium, has been funded by the Snow Foundation and the Ellie White Foundation, and the Liraglutide trial (Victoza) in the United States is currently supported by the Snow Foundation https://thesnowfoundation.org/clinical-trials. The Eye Hope foundation http://www.eyehopefoundation.org/en and the Alianza de Familias Afectadas por el syndrome de wolfram https://afasw.com/have been funding research projects related with the repurposing of GLP-1 analogs for the treatment of Wolfram syndrome among others. The Association Syndrome de Wolfram (https://www.association-du-syndrome-de-wolfram.org/) organizes a family day so that families can get together and share their experiences in coping with the disease. This also provides a way for obtaining mutual support. Additionally, researchers, doctors, and psychologists are invited to provide useful information, namely, about new research and clinical trials.

### Amyotrophic Lateral Sclerosis

#### Clinical Features and Genetic Cause

**FIGURE 4:**
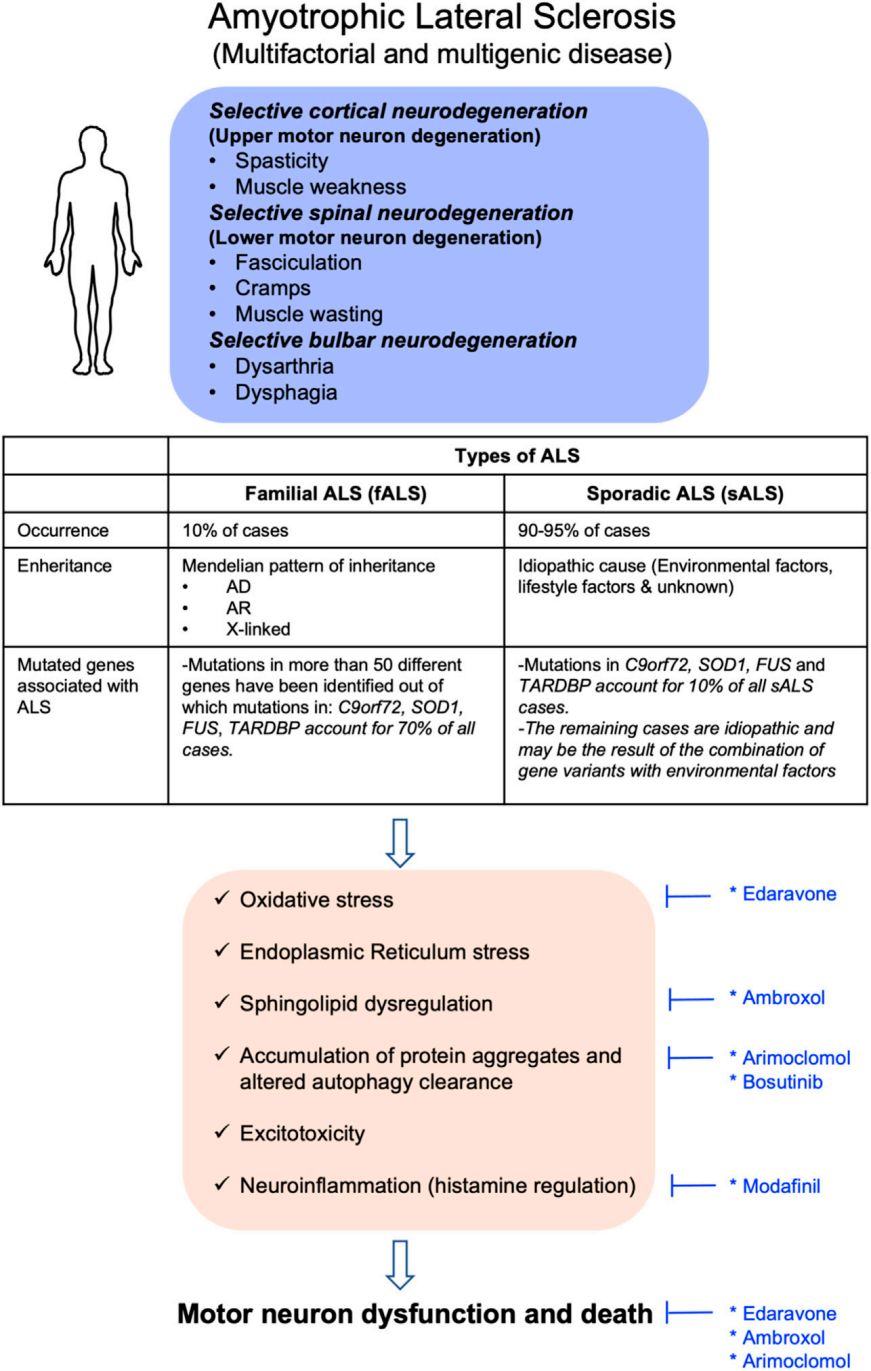
Clinical manifestations and molecular disease mechanisms in amyotrophic lateral sclerosis. This pathology may have familial origin (fALS) or sporadic origin (sALS). The first has Mendelian inheritance, while the second has mostly idiopathic causes. In both forms of ALS, diverse cellular alterations exist resulting in motor neuron dysfunction and death which cause the clinical manifestations of the disease. Repurposed drugs with and without orphan designation for ALS are highlighted in blue along with the cellular dysfunctions or clinical manifestations that they tackle. AD, autosomal dominant; AR, autosomal recessive; *C9orf72,* gene encoding guanine nucleotide exchange; *FUS*, gene encoding the RNA-binding protein FUS; SOD1, gene encoding superoxide dismutase 1; and *TARDBP,* gene encoding TAR DNA-binding protein 43, TDP43.

Amyotrophic Lateral Sclerosis (ALS) is a devastating, highly heterogeneous, rare motor neuron disease with an estimated prevalence in Europe of 2–3 per 100,000 individuals (Logroscino et al., 2010). The disease is characterized by progressive and selective degeneration of the upper motor neurons (projecting from the cortex to the brainstem or the spinal cord) which causes spasticity and muscle weakness and lower motor neurons (projecting from the spinal cord or the brain stem into the muscle) that causes fasciculation, cramps, and muscle wasting (Ravits et al., 2007a; Ravits et al., 2007b; Hardiman et al., 2017; Gromicho et al., 2020). Defects in neuromuscular junctions resulting in skeletal muscle denervation and progressive muscle atrophy have also been described (Cappello and Francolini, 2017). The onset of the disease usually occurs in adulthood during the sixth decade of life, and most ALS patients die from respiratory insufficiency within 2–3 years of symptom onset (Renton et al., 2014; Hardiman et al., 2017). There is currently no treatment to cure the disease. In 60% of the patients ALS has spinal onset, but in some individuals, the disease has bulbar onset that is characterized by dysarthria and dysphagia, while muscle weakness, spasticity, dysarthria, and dysphagia are the most common motor manifestations in ALS; a high proportion of the affected individuals also present cognitive and behavioral impairment (Phukan et al., 2012; Elamin et al., 2013; Al-Chalabi et al., 2017; Hardiman et al., 2017; Matilla-Duenas et al., 2017). ALS is a complex heterogeneous and multifactorial polygenic disorder with a Mendelian pattern of inheritance in 10% of the cases (familial ALS, fALS), and with no evident family history in the remaining cases (sporadic ALS, sALS) (Alsultan et al., 2016). Mostly all fALS instances are inherited in an autosomal dominant way; however, autosomal recessive and X-linked forms also exist. It has been proposed that ALS has oligogenic inheritance (meaning that a phenotypic trait is caused by mutations in more than one gene) and genetic pleiotropy (meaning that a single gene has multiple phenotypic manifestations). Indeed, mathematical models based on population-based registries suggested that ALS patients carry several risk variants that interact with environmental factors predisposing people to the disease (Hardiman et al., 2017). Accordingly, mutations in more than 50 different genes have been identified as being associated with the disease (Mejzini et al., 2019). Mutations in four of those genes, namely, *C9orf72* (encoding guanine nucleotide exchange C9orf72), *SOD1* (encoding superoxide dismutase 1), *FUS* (encoding the RNA binding protein FUS), and *TARDBP (*encoding TAR DNA-binding protein 43, TDP43) account for around 70% of all fALS cases (Al-Chalabi et al., 2017; Hardiman et al., 2017), and were also found to be present in 10% of the sALS cases. In the remaining cases, the actual cause of the disease remains unclear, but it has been proposed that exposure to certain environmental insults and lifestyle factors may influence the development of this disease (Bradley et al., 2013; Wang et al., 2017) (Figure 4). The full list of ALS-associated genes can be found in the study by Mejzini et al., (2019).

### Molecular Phenotype

The pathophysiological mechanisms of the disease are not well understood; however, it is known that aggregation and accumulation of ubiquitylated protein inclusions in the cytosol of motor neurons are a neuropathological hallmark of ALS (Hardiman et al., 2017). Interestingly, in around 97% of ALS patients, these aggregates are constituted of TDP43 protein (even in the absence of *TARDBP* mutations). In some more rare cases, these protein aggregates are constituted of other mutated proteins, such as SOD1. Despite the protein aggregates being the hallmark of ALS, it has been proposed that the high–molecular weight protein complexes (that are present before the aggregate formation) might, in fact, be the real mediators of cell toxicity (Ross and Poirier, 2005).

Multiple factors may contribute to neuronal damage in ALS. Indeed, a large number of cellular alterations have been detected which, in most cases, are the consequence of the identified mutations. Briefly, impaired protein homeostasis and altered autophagy, aberrant RNA metabolism, increased oxidative stress, mitochondrial dysfunction, impaired DNA repair, dysregulated endosomal and vesicle transport, neuroinflammation, glial dysfunction, axonopathy, hyperexcitability, and oligodendrocyte degeneration are the main cellular disturbances detected in ALS (Rowland and Shneider, 2001; Hardiman et al., 2017; Mejzini et al., 2019) (Figure 4). For a detailed description of these mechanisms and their associated genes, refer to the articles by Hardiman et al. (2017) and Mejzini et al. (2019).

### Approved and Non-Approved Orphan-Designated Repurposed Drugs for Amyotrophic Lateral Sclerosis

Currently two orphan-designated drugs received marketing authorization for the treatment of ALS. The first one, riluzole, received this authorization in the United States and Europe, while the second, edaravone, was only authorized by the FDA since the marketing authorization request to the EMA was withdrawn by the developing company in May 2019 (https://www.ema.europa.eu/en/documents/medicine-qa/questions-answers-withdrawal-marketing-authorisation-application-radicava-edaravone_fr.pdf). Riluzole is not a repurposed drug but an anti-glutamatergic compound, specifically developed for the treatment of ALS. It is shown to slow muscle strength deterioration in ALS patients and prolong their survival (Bensimon et al., 1994; Lacomblez et al., 1996a; Lacomblez et al., 1996b; Bensimon et al., 2002).

On the other hand, edaravone is a free radical scavenger that lowers neuronal damage and was initially approved in Japan for the treatment of acute cerebral infarction (Edaravone Acute Infarction Study, 2003). Administration of this molecule soon after the onset of the symptoms improved motor function and decreased SOD1 deposition in rodent ALS models (Ito et al., 2008; Aoki et al., 2011). In a phase II clinical trial with ALS patients, edaravone was relatively safe and well tolerated, and slowed the progression in motor dysfunction (Yoshino and Kimura, 2006). An initial phase III trial, however, failed to demonstrate the efficacy of edaravone to delay the disease progression (Abe et al., 2014). Nevertheless, a post hoc analysis of this trial showed that patients with early symptoms were more prone to respond to the treatment (Takei et al., 2017). Based on these results, an additional phase III clinical trial was performed in which the recruitment criteria were modified in order to work with a more clinically homogeneous patient population who were more likely to respond to the treatment (Writing and Edaravone, 2017). In this study, edaravone was shown to be effective in slowing down the decline in motor function in ALS individuals, fulfilling the criteria identified in the post hoc analysis of the previous phase III study, but not in a wider ALS population, who did not meet this criterion (Writing and Edaravone, 2017). Since all these clinical studies were performed in Japan, and the ALS genetics may vary between populations (Hardiman et al., 2017; Mejzini et al., 2019), further studies are needed to confirm the extrapolability of these findings to other ethnicities. Interestingly, a recent analysis of post-marketing clinical outcomes of edaravone in different countries showed that the drug induced a moderate reduction in disease progression in Kuwait and Korea, while no beneficial effects were reported in Italy and Israel (Ortiz et al., 2020). Further post-marketing reports on the clinical outcomes of edaravone administration are still needed to get a better view of the effectiveness of this drug. Despite riluzole and edaravone being available for the treatment of some ALS patients, there is currently no therapy that can benefit all of them. Therefore, further disease-modifying treatments are needed to handle this life-threatening disease.

Accordingly, because of preclinical and clinical research efforts, more than 30 additional repurposed and non-repurposed substances have received orphan designation for ALS without being yet authorized (https://www.orpha.net/consor/cgi-bin/index.php). Arimoclomol and ambroxol are only two examples of orphan-designated repurposed drugs.

Arimoclomol is a drug originally developed for the treatment of diabetic neuropathy and insulin resistance (Kurthy et al., 2002). This compound is a hydroxylamine derivative that works as a heat shock protein (Hsp) co-inducer in conditions of cellular stress (Hargitai et al., 2003). Treatment with this molecule results in the upregulation of several Hsps, including Hsp60, HsP70, Hsp90, and Grp94 (Vigh et al., 1997). Modulation of the Hsp response was shown to be beneficial for diseases associated with protein aggregation since it reduces the protein aggregation itself and diminishes cellular stress and apoptosis (Kalmar et al., 2005; Kalmar et al., 2014). Related with ALS, arimoclomol was shown to improve limb strength, neuron survival, and life span of SOD1^G93A^ mice, a fALS model, while reducing the abundance of ubiquitin-positive aggregates in the motor neurons (Kieran et al., 2004; Kalmar et al., 2008). In addition, arimoclomol was shown to be safe and well tolerated in a double-blind, placebo-controlled trial performed with rapidly progressive SOD1-mutant ALS patients (Benatar et al., 2018). This initial study also suggested that the molecule could have therapeutic benefit for these patients (Benatar et al., 2018). Unfortunately, a randomized, placebo-controlled, phase III clinical trial with arimoclomol (ORARIALS-01 phase III trial, NCT03491462), that was started in 2018 and whose results were much awaited, failed to improve motor function and patient survival upon chronic treatment, as well as the ability to perform daily tasks, time to permanent assisted ventilation, and changes in lung function (defined as secondary endpoints of the study) https://alsnewstoday.com/news-posts/2021/05/11/arimoclomol-fails-phase-3-als-trial-does-not-show-efficacy-per-topline-data/).

Ambroxol, a beta-glucocerebrosidase 2 (GBA2) inhibitor originally indicated as a generic expectorant and mucolytic drug to treat respiratory tract infectious disorders (Nobata et al., 2006), has been recently found to be a potential drug candidate for ALS treatment. In a fALS transgenic mouse model, the SOD1^G86R^ mouse, this drug delayed the disease onset, improved motor function, and rescued neuronal death by regulating the glycosphingolipid metabolism, a pathway that is importantly altered in the CNS of these mice and ALS patients (Dodge et al., 2015; Henriques et al., 2015; Henriques et al., 2017; Bouscary et al., 2019; Bouscary et al., 2020). Despite these promising results in mice, clinical trials are still needed to evaluate the safety and effectiveness of this drug in ALS patients. Table 4 provides additional information about the aforementioned drugs, including pharmaceutical companies or academic institutions involved in designing and running the clinical trials, and whether the drugs received orphan designation from the EMA and/or the FDA, and the sponsors that made the orphan designation request.

**TABLE 4:** List of the repurposed drugs, with and without orphan designation or drug marketing authorization for amyotrophic lateral sclerosis (ALS) mentioned in this article. The sponsors (entities involved in making the orphan designation request to the EMA or the FDA) and the public or private organizations involved in designing and running the clinical trials are detailed. N/A = non-applicable.

Drug name	Orphan drug designation for ALS?	Sponsor	Information about the sponsors	Drug marketing authorization for ALS?	Original indication of the drug	Mechanism of action and preclinical findings	Outcome of the clinical trials	Entities involved in designing and running the clinical trials	References
Edaravone MCI-186 (Radicava^®^, Univone^®^)	Yes: EMA, 2014 and 2015; FDA, 2015	*Europe:* 2014 Orphan drug designation given to Treeway B.V, Netherlands *. 2015 Orphan drug designation given to Mitsubishi Tanabe Pharma Europe Ltd., United Kingdom and then transferred to Mitsubishi Tanabe Pharma GmbH, Germany in 2019 § *US:* Mitsubishi Tanabe Pharma GmbH, Germany §	*Clinical-stage pharmaceutical company. §Japanese pharmaceutical company	Yes	Acute ischemic stroke	Free radical scavenger. This drug improved motor function in rodent ALS models	In a phase II trial, the drug was found to be safe and well tolerated. In a phase III trial, edaravone slowed down the decline in motor function only in a subset of ASL patients	Trials sponsored by Mitsubishi Tanabe Pharma Corporation; or Loma Linda University, or Mitsubishi Tanabe Pharma Development America, Inc.; or Isfahan University of Medical Sciences, Iran; or Mitsubishi Tanabe Pharma Corporation, Mitsubishi Tanabe Pharma Development America, Inc.; or Ruijin Hospital, China, in collaboration with Temple University, Thomas Jefferson, University, the University of Southern California, Northwestern University, and various academic institutions and hospitals from the US, Canada, France, Italy, Japan, Iran, and China	Yoshino and Kimura. (2006); Ito et al. (2008); Aoki et al. (2011)
									Abe et al. (2014); Writing and Edaravone. (2017); Ortiz et al. (2020)
									https://clinicaltrials.gov/ct2/show/NCT00424463?term=edaravone&cond=Amyotrophic+Lateral+Sclerosis&draw=2&rank=1
									https://clinicaltrials.gov/ct2/show/NCT00330681?term=edaravone&cond=Amyotrophic+Lateral+Sclerosis&draw=2&rank=2
									https://clinicaltrials.gov/ct2/show/NCT00415519?term=edaravone&cond=Amyotrophic+Lateral+Sclerosis&draw=1&rank=3
									https://clinicaltrials.gov/ct2/show/NCT04577404?term=edaravone&cond=Amyotrophic+Lateral+Sclerosis&draw=2&rank=4
									https://clinicaltrials.gov/ct2/show/NCT01492686?term=edaravone&cond=Amyotrophic+Lateral+Sclerosis&draw=2&rank=5
									https://clinicaltrials.gov/ct2/show/NCT04097158?term=edaravone&cond=Amyotrophic+Lateral+Sclerosis&draw=2&rank=6
									https://clinicaltrials.gov/ct2/show/NCT03272802?term=edaravone&cond=Amyotrophic+Lateral+Sclerosis&draw=2&rank=7
									https://clinicaltrials.gov/ct2/show/NCT04259255?term=edaravone&cond=Amyotrophic+Lateral+Sclerosis&draw=2&rank=8
									https://clinicaltrials.gov/ct2/show/NCT04176224?term=edaravone&cond=Amyotrophic+Lateral+Sclerosis&draw=2&rank=9
									https://clinicaltrials.gov/ct2/show/NCT04254913?term=edaravone&cond=Amyotrophic+Lateral+Sclerosis&draw=2&rank=10
									https://clinicaltrials.gov/ct2/show/NCT04391361?term=edaravone&cond=Amyotrophic+Lateral+Sclerosis&draw=2&rank=11
									https://clinicaltrials.gov/ct2/show/NCT04569084?term=edaravone&cond=Amyotrophic+Lateral+Sclerosis&draw=2&rank=12
Arimoclomol	Yes: EMA, 2006; US, 2005	Europe: Orphan designation given to Orphazyme A/S, Denmark * in 2006, and then transferred to Wainwright Associates Ltd., United Kingdom, in 2012 & (this company changed the name to PharmaLex, United Kingdom in 2016). US: Orphazyme A/S, Denmark *	*Biopharmaceutical company &Consulting	No	Diabetic neuropathy and insulin resistance	Heat shock protein (Hsp) co-inducer in conditions of cellular stress. The molecule improved limb strength, neuron survival, and life span in a mouse model of familial ALS and reduced the abundance of ubiquitin-positive aggregates in the motor neurons	The drug was shown to be safe and well tolerated in a double-blind, placebo-controlled trial that also suggested potential beneficial effects for the patients. A follow-up phase III trial failed to meet its primary endpoints (improvement in motor function and patient survival upon chronic drug administration)	Trials sponsored by the University of Miami; or Orphazyme (pharmaceutical company); or CytRx corporation (pharmaceutical company) in collaboration with 29 academic institutions and hospitals from the US, Canada, Belgium, France, Spain, Germany, Italy, Poland, the Netherlands, Sweden, Switzerland, and the United Kingdom.	Kieran et al. (2004); Kalmar et al. (2008); Benatar et al. (2018)
									https://clinicaltrials.gov/ct2/show/NCT00706147?term=Arimoclomol&cond=Amyotrophic+Lateral+Sclerosis&draw=2&rank=1
									https://clinicaltrials.gov/ct2/show/NCT03836716?term=Arimoclomol&cond=Amyotrophic+Lateral+Sclerosis&draw=2&rank=3
									https://clinicaltrials.gov/ct2/show/NCT03491462?term=Arimoclomol&cond=Amyotrophic+Lateral+Sclerosis&draw=2&rank=4
									https://clinicaltrials.gov/ct2/show/NCT00244244?term=Arimoclomol&cond=Amyotrophic+Lateral+Sclerosis&draw=2&rank=5
Ambroxol (MucoAngin^®^)	Yes: EMA, 2017	Europe: Spedding Research Solutions SAS, France	Micro biopharma company (Micro TPE) with consulting activities	No	Expectorant and mucolytic drug	Regulates the sphingolipid pathway and glucocerebrosidase activity. In a mouse model of familial ALS, the drug improved motor function and prolonged mice survival Further clinical studies are required to prove its efficacy and safety in ALS patients	No clinical trials were performed with this drug	N/A	Dodge et al. (2015); Henriques et al. (2015); Henriques et al. (2017); Bouscary et al. (2019); Bouscary et al. (2020)
Pimozide (ORAP^®^)	No	N/A	N/A	No	Drug indicated to treat chronic psychosis, Tourette syndrome, and tics	Neuroleptic. The acute administration of this drug improved motor function in *C. elegans*, zebrafish, and mouse models of ALS, but its chronic administration failed to improve motor function in two different mouse ALS models and induced some toxic effects	An unblinded clinical study with ALS patients suggested that the drug may reduce disease progression. The drug was shown to be safe and well tolerated in a subsequent pilot randomized, double-blinded, placebo-controlled trial. A phase II trial to evaluate the effectiveness of the drug is still ongoing	Trials Sponsored by the University of Calgary in collaboration with ALS Canada (patient association), Brain Canada (patient association), Hotchkiss Brain Institute, the University of Calgary, and several academic institutions and hospitals of Canada	Patten et al. (2017) Pozzi et al. (2018)
									https://clinicaltrials.gov/ct2/show/NCT02463825?cond=Amyotrophic+Lateral+Sclerosis&draw=5&rank=359
									https://clinicaltrials.gov/ct2/show/NCT03272503?cond=Amyotrophic+Lateral+Sclerosis&draw=4&rank=240
									https://clinicaltrials.gov/ct2/show/NCT02463825?term=pimozide&cond=Amyotrophic+Lateral+Sclerosis&draw=1&rank=2
Bosutinib (Bosulif^®^)	No	N/A	N/A	No	Indicated for the treatment of chronic myeloid leukemia	Tyrosine kinase inhibitor *In vitro* and vivo preclinical studies with different ALS models showed that this drug may have neuroprotective effects	An open-label multicenter, phase I, dose-escalating clinical trial with bosutinib in ALS patients has recently been launched to assess the safety, tolerability, and efficacy of the molecule	This trial is sponsored by Kyoto University, in collaboration with Tokushima University, Kitasato University, Tottori University, and Pfizer (Pharmaceutical company)	Imamura et al. (2017); Osaki et al. (2018); Imamura et al. (2019)
									https://clinicaltrials.gov/ct2/show/NCT04744532?cond=Amyotrophic+Lateral+Sclerosis&draw=13&rank=287
Modafinil (Provigil^®^)	No	N/A	N/A	No	Indicated for treatment of hypersomnolence and narcolepsy	This drug elevates histamine levels in the neocortex and the hypothalamus	A placebo-controlled study showed that this drug is safe and well tolerated and it decreases fatigue in ALS patients	This trial was sponsored by New York State Psychiatric Institute, US	Fiscon et al. (2021) Carter et al. (2005); Rabkin et al. (2009)
						An *in silico drug* repurposing screening identified this drug as having potential beneficial effects in ALS	Further exploratory studies are required to evaluate additional beneficial effects of the drug in the management of the disease		https://clinicaltrials.gov/ct2/show/NCT00614926

The following websites were consulted to build up this table. Community Register of orphan medicinal products from the European commission: https://ec.europa.eu/health/documents/community-register/html/reg_od_act.htm?sort=a.. Orphan Drug Designations and Approvals from the FDA: https://www.accessdata.fda.gov/scripts/opdlisting/oopd/.ClinicalTrial.gov to retrieve the list of clinical trials for ALS: https://clinicaltrials.gov/ct2/results?cond=Amyotrophic+Lateral+Sclerosis&term=&cntry=&state=&city=&dist=.

### Additional Drug Repurposing–Based Therapeutic Strategies Under Investigation

Besides the molecules described above, a large number of additional repurposed drugs are currently being tested as potential therapeutic agents for ALS. Between them, neuroleptics have been identified as lead compounds for the management of the disease. Recently Patten et al (2017) performed a phenotypic drug screening of an approved drug compound library containing 3,850 molecules on *C. elegans* transgenics-expressing mutant TDP-43 (a protein mutated in ALS patients). Since the worms expressing the mutated protein exhibit paralysis phenotypes, this model allows the identification of compounds with beneficial effects in motor function. From the 24 compounds identified as potentially beneficial, 13 were neuroleptics. The positive hits were retested in worms and in zebrafish, expressing mutated TDP-43, FUS, and SOD1. These studies identified pimozide, an FDA-approved neuroleptic used to treat chronic psychosis, Tourette syndrome, and tics (Shapiro et al., 1987), as the drug with the strongest beneficial effect in motor function in these two models and a rodent model of fALS—the SOD1^G37R^ mouse (Patten et al., 2017). Despite these promising results, a recent study with two other ALS mouse models showed that the chronic administration of pimozide does not attenuate motor and pathological deficits and, in some cases, had deleterious effects (Pozzi et al., 2018). Regarding the effect of the drug in humans, an initial clinical study analyzing the effect of pimozide in ALS patients compared to other potentially neuroprotective compounds suggested that this drug may reduce the patient’s disease progression (Szczudlik et al., 1998). Since this trial was unblinded, a factor that may importantly influence the results, two additional trials were performed: a pilot randomized, double-blinded, placebo-controlled trial with ALS patients to assess the safety and tolerability of pimozide, and phase II randomized, placebo-controlled, double-blinded, multi-centre clinical trial of pimozide in 100 ALS patients to evaluate the effectiveness of the drug in slowing ALS progression. The former showed that the drug is safe and well tolerated in doses up to 4 mg/day (Patten et al., 2017), while the latter, initiated in October 2017, has not been finished yet.

An inducible drug repurposing screening using pluripotent stem cell (iPSC)–derived motor neurons from an ALS patient with an SOD1 mutation identified bosutinib, a tyrosine kinase inhibitor indicated for the treatment of chronic myeloid leukemia, as a drug with potential therapeutic benefits for ALS (Imamura et al., 2017). This molecule was not only beneficial for the cells mentioned before but also increased the survival of iPSC-derived motor neurons from patients with sALS or other forms of fALS (Imamura et al., 2017), and the contractability of iPSC-derived skeletal muscle cells from ALS patients (Osaki et al., 2018). In addition, bosutinib modestly extended the survival of an ALS mouse model with SOD1 mutation (Imamura et al., 2017). Based on these promising findings, an open-label, multicenter, phase I, dose-escalating clinical trial with bosutinib in ALS patients (iDReAM study) has recently been designed to assess the safety, tolerability, and efficacy of the molecule (Imamura et al., 2019). No results are yet available for this trial.

Besides these efforts*, in silico* approaches are currently being actively used for the identification of repurposed drug candidates with potential beneficial effects in ALS. These strategies that exploit current knowledge on disease-associated genes and disease mechanisms, protein–protein interactions, signaling networks, and drug-target interactions allow to save time and resources on the identification of candidate compounds. Accordingly, Fiscon et al. (2021) exploited SAveRUNNER, a recently developed network-based algorithm for drug repurposing, which quantifies the proximity of disease-associated genes to drug targets to identify drug candidates for ALS. This approach allowed to identify 403 repurposable drugs that were strongly associated with the disease. Most of these compounds belonged to drug families already identified as having disease-modifying potential, but some were non-customary ALS drugs (Fiscon et al., 2021). Among the latter, modafinil, a compound that elevates histamine levels in the neocortex and the hypothalamus and which is currently indicated for the treatment of hypersomnolence and narcolepsy (Ishizuka et al., 2010), was identified as the drug with the highest predictive score for ALS alone or combined with other drugs that are currently being tested in clinical trials (Fiscon et al., 2021). In the past, modafinil was proposed as a drug used to treat fatigue in ALS patients. An open-label, control study and a placebo-controlled trial with modafinil showed that this drug is safe and well tolerated and may, indeed, decrease fatigue in ALS patients (Carter et al., 2005; Rabkin et al., 2009). These results and the ones from the *in silico* prediction study (Fiscon et al., 2021) point to modafinil as a promising drug for ALS treatment whose beneficial effect needs to be proved in further clinical trials.

The main characteristics of the drugs mentioned above are summarized in Table 4.

### Amyotrophic Lateral Sclerosis Patient Associations and Foundations

There are a large number of ALS patient associations and foundations that are compiled in the North database (https://rarediseases.org/rare-diseases/amyotrophic-lateral-sclerosis/). The ALS association (https://www.als.org/), ALS Canada (https://www.als.ca/), the International Alliance of ALS/MND associations (https://www.als-mnd.org/), and ALS Liga België Vzw–Ligue SLA Belgique Asbl (https://als.be/fr/qui-sommes-nous) are just a few examples of non-profit organizations that provide assistance to people with ALS, coordinate multidisciplinary care, and fund research programs all over the word to discover treatments and a cure for ALS.

## Conclusion

The four rare neurodegenerative diseases taken as example in this study clearly highlight the limited availability of approved therapies for the management of rare pathologies, harnessing the importance of drug repurposing–based approaches to fill these gaps. Indeed, drug repurposing appears to be a cost- and time saving procedure in the research and development of orphan drugs. The identification of repurposed drugs with potential therapeutic benefit involves experimental or *in silico* approaches that depend on a good understanding of the molecular disease mechanisms, highlighting the key role of fundamental research in the process. During the last years, the utilization of patient stem cell–based high-throughput screening approaches and *in silico* prediction tools such as network mapping, genome-wide association studies, (GWAS) and rare variant association studies (RVAS) significantly accelerated the identification of candidate drugs for disease treatment. However, despite these very good advances that helped identify promising drugs for the treatments of different rare diseases, including HD, FRDA, Wolfram syndrome, and ALS, there is still an important translational gap between the volume of preclinical and clinical research conducted and the number of repurposed approved drugs. This is the consequence of several factors, for example, the complexity of organizing clinical trials in rare diseases due to reduced patient number, their wide geographical distribution, their short life span, and the severity of the diseases that complicate the study design, the determination of the relevant clinical endpoints, and the ethical concerns in introducing a placebo group in the trial which, if present, may discourage the patients to participate in the study, and if absent will reduce the validity of the trial. Moreover, the important rate of late-stage failure of clinical studies, as observed for the rare neurodegenerative diseases described in this article is another important factor contributing to the reduced number of repurposed approved drugs. Indeed, HD, Wolfram syndrome, FRDA, and ALS are largely heterogenous diseases with varying severity. Clinical trials performed in large heterogenous patient cohorts often failed to demonstrate the benefits of the drugs under investigation despite very promising preclinical and pilot clinical data. This points to the need for a better clinical trial design to evaluate drug effects in specific patient cohorts as highlighted by several post hoc data analyses.

Taking all this into consideration, further collaborative initiatives between pharmaceutical companies, small- and medium-sized enterprises, academic researchers, patient associations, and regulatory authorities are needed, which not only accelerate the identification and validation of repurposed compounds but also improve the design and organization of clinical trials. Indeed, a more systematic interaction between the clinical trial organizers and the regulatory authorities in terms of scientific advice or protocol assistance appears as the key to ameliorate the trial design by defining valid endpoints, the minimum number of patients to be enrolled, and the study format (e.g., placebo-controlled trial or N-of-1 trials). All this is expected to improve the rate of the approval of new candidate compounds and accelerate their access to the affected patients.

In conclusion, drug repurposing appears as an excellent platform to accelerate drug discovery and availability of therapeutics not only for rare neurodegenerative diseases but also for other rare pathologies. This approach can be fostered by the implementation of better economic incentives by the governments in collaboration with public–private partnerships.

## Glossary

ALS: Amyotrophic lateral sclerosis
BDNF: Brain-derived neurotrophic factor
CDRC: CURE Drug Repurposing Collaboratory
CNS: Central nervous system
CPAD: Critical path for Alzheimer’s disease
CPP: Critical Path for Parkinson’s
CPTA: Critical Path to Therapeutics for Ataxia
EMA: European Medicines Agency
ER: Endoplasmic reticulum
ERN-RND: European Reference Network for Rare Neurological Disorders
EU: European Union
EHA: European Huntington’s Association
EURORDIS: European Organization for Rare Diseases
FA-ICD: Friedreich’s Ataxia-Integrated Clinical Database
fALS: familial ALS
FDA: Food and Drug Administration
FXN: Frataxin
FRDA: Friedreich’s ataxia
GBA2: Beta-glucocerebrosidase 2
GLP-1: Glucagon-like peptide-1
HD: Huntington’s disease
HDAC: Histone deacetylase
HD-RSC: Huntington’s Disease Regulatory Science Consortium
HDSA: Huntington’s Disease Society of America
Hsp: Heat shock protein
HSC: Huntington’s Society of Canada
HTT: Huntington
IRDiRC: International Rare Diseases Research Consortium
iPSC: Inducible pluripotent stem cell
mHTT: mutated Huntington
MRC: Medical Research Council
MRI: Magnetic resonance imaging
mTOR: Mammalian target of rapamycin
NCATS: National Center for Advancing Translational Sciences
NGS: Next-generation sequencing
NIH: National Institutes of Health
NORD: National Organization of Rare Disorders
ODA: Orphan Drug Act
R&D: Research and Development
sALS: Sporadic ALS
SME: Small- and medium-sized enterprise
VMAT: Vesicular monoamine transporter
UK: United Kingdom
US: United States
